# LASS2 enhances chemosensitivity to cisplatin by inhibiting PP2A-mediated β-catenin dephosphorylation in a subset of stem-like bladder cancer cells

**DOI:** 10.1186/s12916-023-03243-5

**Published:** 2024-01-09

**Authors:** Hongjin Shi, Zhiyong Tan, Bowen Duan, Chunming Guo, Chong Li, Ting Luan, Ning Li, Yinglong Huang, Shi Chen, Jixian Gao, Wei Feng, Haole Xu, Jiansong Wang, Shi Fu, Haifeng Wang

**Affiliations:** 1 Department of Urology, the Second Affiliated Hospital of Kunming Medical University, Kunming, China; 2 Yunnan Clinical Medical Center of Urological Disease, Kunming, China; 3 Kunming Medical University, Kunming, China; 4 School for Life Science, Yunnan University, Kunming, China; 5 Institute of Biophysics, Chinese Academy of Sciences, Beijing, China

**Keywords:** Bladder cancer, Cancer stem cell, Drug resistance, Ceramide, Cisplatin sensitivity, β-catenin

## Abstract

**Background:**

The benefits of first-line, cisplatin-based chemotherapy for muscle-invasive bladder cancer are limited due to intrinsic or acquired resistance to cisplatin. Increasing evidence has revealed the implication of cancer stem cells in the development of chemoresistance. However, the underlying molecular mechanisms remain to be elucidated. This study investigates the role of LASS2, a ceramide synthase, in regulating Wnt/β-catenin signaling in a subset of stem-like bladder cancer cells and explores strategies to sensitize bladder cancer to cisplatin treatment.

**Methods:**

Data from cohorts of our center and published datasets were used to evaluate the clinical characteristics of LASS2. Flow cytometry was used to sort and analyze bladder cancer stem cells (BCSCs). Tumor sphere formation, soft agar colony formation assay, EdU assay, apoptosis analysis, cell viability, and cisplatin sensitivity assay were used to investigate the functional roles of LASS2. Immunofluorescence, immunoblotting, coimmunoprecipitation, LC–MS, PCR array, luciferase reporter assays, pathway reporter array, chromatin immunoprecipitation, gain-of-function, and loss-of-function approaches were used to investigate the underlying mechanisms. Cell- and patient-derived xenograft models were used to investigate the effect of LASS2 overexpression and a combination of XAV939 on cisplatin sensitization and tumor growth.

**Results:**

Patients with low expression of LASS2 have a poorer response to cisplatin-based chemotherapy. Loss of LASS2 confers a stem-like phenotype and contributes to cisplatin resistance. Overexpression of LASS2 results in inhibition of self-renewal ability of BCSCs and increased their sensitivity to cisplatin. Mechanistically, LASS2 inhibits PP2A activity and dissociates PP2A from β-catenin, preventing the dephosphorylation of β-catenin and leading to the accumulation of cytosolic phospho-β-catenin, which decreases the transcription of the downstream genes ABCC2 and CD44 in BCSCs. Overexpression of LASS2 combined with a tankyrase inhibitor (XAV939) synergistically inhibits tumor growth and restores cisplatin sensitivity.

**Conclusions:**

Targeting the LASS2 and β-catenin pathways may be an effective strategy to overcome cisplatin resistance and inhibit tumor growth in bladder cancer patients.

**Supplementary Information:**

The online version contains supplementary material available at 10.1186/s12916-023-03243-5.

## Background

Bladder cancer is the tenth most common cancer worldwide and accounts for an estimated 91,893 new cases and 42,973 deaths in China in 2022 [1, 2]. Although bladder cancer is early detection and recurrence monitoring by some DNA methylation-based urine assays. However, the 5-year overall survival rate for patients with muscle-invasive bladder cancer is poor [3]. Standard treatment for muscle-invasive bladder cancer (MIBC) typically involves cisplatin-based chemotherapy. However, nearly half of MIBC patients are intrinsically resistant to cisplatin-based chemotherapy or develop resistance to it [4].

Cisplatin is a simple compound that consists of two amides, two chlorides, and a platinum atom. It works by binding to DNA and creating intrastrand crosslink adducts that generate an apoptotic signal [5]. Mechanisms of cisplatin resistance are multifaceted and include increased drug efflux, increased DNA damage repair, and inhibition of cell death pathways [6]. Currently, it is known that chemoresistant cells share characteristics with stem-like subpopulations that often survive after chemotherapy and are responsible for cancer relapse [7]. Wnt/β-catenin signaling has been demonstrated to be associated with cisplatin resistance [8] and cancer stem cells (CSCs) [9]. Therefore, exploring strategies that target CSCs and Wnt/β-catenin signaling can help overcome cisplatin resistance.

Ceramides, which are precursors to various sphingolipids, are frequently involved in regulating apoptosis pathways [10]. Several studies have revealed the direct involvement of ceramides in the regulation of chemotherapy resistance [11] and CSCs [12]. Ceramide synthase (CERS/LASS) is primarily engaged in ceramide synthesis, and six kinds of LASS proteins have been identified in human [13]. Previous studies have shown that LASS1 plays important roles in the regulation of gemcitabine/doxorubicin-induced caspase activation and cell death in head and neck squamous cell carcinoma [14], LASS6 enhanced the killing effect of sorafenib and vorinostat on colon cancer cells [15]. LASS2, also known as ceramide synthase 2 (CERS2), is one of six ceramide synthases (CERS1-6) in humans and selectively produces C22-C24 ceramides [16]. It has been identified as a cancer suppressor gene in some cancers [17–19]. For example, a recent typical study found that approximately 50% of LASS2 null mice developed pheochromocytoma by 13 months, possibly due to defects in apoptosis [20]. We recently discovered that upregulation of LASS2 increased cisplatin sensitivity in bladder cancer cells [21]. Nonetheless, little is known about the mechanisms of LASS2 in regulating chemotherapy resistance in bladder cancer.

This study aims to investigate the associations between LASS2 and cisplatin resistance in bladder cancer stem cells (BCSCs) and uncover the underlying molecular mechanisms, ultimately evaluating the role of LASS2 as a chemosensitizing target for bladder cancer. Our results shed light on the role of LASS2 in regulating the β-catenin pathway and provide novel strategies for overcoming cisplatin resistance in bladder cancer.

## Methods

### Patients and specimens

Clinical information and tissue specimens were collected from 104 bladder cancer patients who underwent radical cystectomy at our center between 2014 and 2015. Among these patients, a subgroup analysis was conducted on 44 patients who received cisplatin-based adjuvant chemotherapy. The clinical information, including age, sex, grade, and TNM stage, was obtained from medical records. The overall survival (OS) time was measured from the date of diagnosis to the date of the last follow-up or death. The expression of LASS2 was determined by immunohistochemistry (IHC) staining and H-Score (as described below). In addition, to analyze the associations between LASS2 expression and chemotherapy response, 45 specimens were collected from MIBC patients who received cisplatin-based neoadjuvant chemotherapy (NAC) (Additional file 1). The specimens were obtained through transurethral resection of bladder tumor (TURBT) before chemotherapy. The chemotherapy regimen used gemcitabine/cisplatin (GC) and was administered based on CSCO guidelines (gemcitabine, 1000 mg/m^2^ d1 and d8; cisplatin 70 mg/m^2^ d2, 21-day cycle for 3 or 4 cycles). The chemotherapy responses were evaluated using the Response Evaluation Criteria in Solid Tumors criteria version 1.1 [22]. Chemosensitivity was defined as patients achieving a complete response (CR) or partial response (PR). Chemoresistance was defined in patients with stable disease (SD) or progressive disease (PD). Clinical information and specimens were obtained after writing informed consent according to a protocol approved by the Ethics Committee of the Second Affiliated Hospital of Kunming Medical University.

### Public database analysis

The expression profiles and clinical data of bladder cancer patients were acquired from The Cancer Genome Atlas (TCGA) portal (https://portal.gdc.cancer.gov). Copy number variation (CNV) data of the TCGA_BLCA dataset were obtained from UCSC Xena (http://xena.ucsc.edu) and processed through GISTIC2.0 [23]. The molecular subtypes of TCGA_BLCA dataset were determined using the BLCAsubtyping R package (https://github.com/cit-bioinfo/BLCAsubtyping) [24]. Furthermore, two datasets (GSE70691 and GSE69795) with sequencing data and clinical information were downloaded from the Gene Expression Omnibus (GEO) and analyzed. The raw RNA-sequencing data were transformed into transcripts per kilobase million (TPM). To explore the relationship between LASS2 and cisplatin sensitivity, the transcriptional data of bladder cancer cell lines and their corresponding cisplatin IC_50_ values were obtained from the Genomics of Drug Sensitivity in Cancer (GDSC) database (https://www.cancerrxgene.org) [25, 26]. The pathway activity score of 10 cancer-related pathways was calculated using reverse phase protein array (RPPA) data of the TCGA_BLCA dataset, which were obtained from The Cancer Proteome Atlas (TCPA) database (https://www.tcpaportal.org) [27, 28]. Specifically, replicate-based normalization RPPA data were median-centered and normalized by the standard deviation across all samples for each component to obtain the relative protein level. The pathway score was then calculated as the sum of the relative protein level of all positive regulatory components minus that of negative regulatory components in a particular pathway. Finally, gene set enrichment analysis (GSEA) was performed based on TCGA_BLCA expression profiles using GSEA 2.0.9 (http://www.broadinstitute.org/gsea/).

### Cell lines and culture

The human cell lines 5637, T24, RT4, J82, SW780, UMUC-3, A549, and SK-OV-3 were obtained from the American Type Culture Collection (Maryland, USA). All cell lines were authenticated by STR profiling and tested for mycoplasma contamination. 5637, T24, SW780, and A549 cells were grown in RPMI-1640 medium supplemented with 10% fetal bovine serum (FBS). RT4, J82, and UMUC3 cells were grown in DMEM/F12 supplemented with 10% FBS. SK-OV-3 cells were grown in McCoy’s 5a medium with 10% FBS. The sorted BCSCs (as described below) were maintained in serum-free DMEM/F-12 supplemented with 20 ng/mL EGF (#PHG0311, Gibco), 20 ng/mL FGF-basic (#13256–029, Gibco), 1% N-2 (#17502001, Gibco), and 2% B-27 (#17504044, Gibco). BCSCs were passaged less than 10 times in vitro. Primary bladder cancer cells (pBCs) were isolated from a chemotherapy-naïve patient. Briefly, fresh bladder tumors were minced into small fragments and digested with 200 U type I collagenase (#07415, STEMCELL Technologies), 0.01% hyaluronidase (#07461, STEMCELL Technologies), and 0.01% DNase I in RPMI 1640 at 37°C for 120 min. Subsequently, cells were filtered through a cell strainer (70 μm), washed, suspended with phosphate-buffered saline (PBS) to yield single cells, or cultured in complete medium for further experiments.

### Construction of cisplatin-resistant cell lines

Parent 5637 cell lines were continuously exposed to an escalating dose of cisplatin (0.5–16 μg/mL) until the IC_50_ of cisplatin reached 5 times that of parental cells. For approximately 6 months, a cisplatin-resistant subclone of 5637 cells (5637-CR) were established. The 5637-CR cells were maintained in RPMI-1640 medium supplemented with 10% FBS and 30 μM cisplatin. The resistance factor was calculated as the ratio of the IC_50_ of the parental cells to that of the resistant cells.

### BCSCs sorting and flow cytometry analysis

To sort BCSCs, a total of 10^5^ 5637-CR cells or isolated tumor cells were resuspended in 200 μL of HBSS with 2% FBS and stained with fluorescein isothiocyanate (FITC)-conjugated anti-CD44 (#75122, CST) and PE-conjugated anti-ALDH1A1 (#65583, CST). Single cells were gated by plotting SSC-A versus FSC-A and confirmed by gating FSC-W versus FSC- H and SSC-A versus SSC-H. Viable cells were identified by negative staining of 7-aminoactinomycin D (A9400, Sigma–Aldrich). Subsequently, the CD44^+^ALDH1A1^+^ and CD44^−^ALDH1A1^−^ populations were assayed and sorted using a FACSArila III flow cytometry system (BD Biosciences, USA) and collected in cold PBS in 5-mL polypropylene tubes. The gating strategies are presented in Additional file 2: Fig. S5A.

For flow cytometry analysis, primary cells or cell lines were dissociated into single cells with trypsin–EDTA. Single-cell suspensions (10^6^/mL) were stained with FITC-CD44 and PE-ALDH1A1 or the same isotype FITC/PE-conjugated antibodies (#5742, #97146, CST) for 30 min. Subsequently, the stained cells were fixed with 4% PFA (BD Biosciences, US) for 20 min. The percentages of CD44^+^ALDH1A1^+^, CD44^−^ALDH1A1^+^, CD44^+^ALDH1A1^−^ and CD44^−^ALDH1A1^−^ cells were analyzed by flow cytometry using a FACSArila III flow cytometry system (BD Biosciences, US). Flow cytometry data were processed using FlowJo software (version 10.7.1).

For apoptosis analysis, 100 μL of single-cell suspensions were prepared with annexin V binding buffer. The cells were then incubated with 5 μL FITC-annexin V and 5 μL PI (BD Biosciences, USA) for 15 min at room temperature in the dark. The annexin V-positive cells were analyzed using a FACSArila III flow cytometry system (BD Biosciences, USA).

### Plasmids and cell transfection

To downregulate the expression of LASS2, two short hairpin RNAs (shRNAs) were cloned into the pLV-EGFP-U6 vector (GeneChem, Shanghai, China) and transfected into bladder cancer cells using Lipofectamine LTX Reagent with Plus Reagent (#15338100, Invitrogen). The sequences (5′-target sequence-stem loop-antisense strand-3′) were as follows: shRNA1: 5′-CCTGCCTTCTTTGGCTATTAC-TTCAAGACG-GTAATAGCCAAAGAAGGCAGG-3′, shRNA2: 5′-ATGGCCGTCATTGTGGATAAA-TTCAAGACG-TTTATCCACAATGACGGCCAT-3′. The plasmid containing the scrambled sequence (5′-CCTAAGGTTAAGTCGCCCTCG-3′) was used as a negative control. LASS2 stable knockdown cells were selected using 2 μg/mL puromycin for culturing for 2 weeks. And the knockdown efficiency was verified by western blot. Two shRNAs were used, and shRNA2, which was named shLASS2 in this paper, was chosen to carry out subsequent experiments because of its higher interference efficiency (Additional file 2: Fig. S3E).

The LASS2 ORF expression plasmid (#RG215300) and PP2A catalytic subunit expression plasmid (#SC321401) with the pCMV6-Ac-GFP vector were obtained from OriGene. siRNAs for GSK3β (sc-35525) and CK1α (sc-29912) were obtained from Santa Cruz. For some experiments, 5 × 10^4^ cells were transfected with 0.1 μg/mL plasmid per well in 12-well dishes using Lipofectamine LTX Reagent with Plus Reagent (#15338100, Invitrogen) for 24 h, and the empty vector was used as a control. The overexpression efficiency was verified by western blot (Additional file 2: Fig. S3E).

### Tumor sphere formation and in vitro extreme limiting dilution assay

Cells from the indicated groups were seeded at a density of 1000 cells per well in a 6-well plate with an ultra-low attachment surface (Corning). Tumor spheres were generated in serum-free DMEM/F-12 and supplemented with 20 ng/mL EGF, 20 ng/mL FGF-basic, 1% N-2, and 2% B-27. After 10 days, photomicrographs of tumor spheres were taken, and the size and number of tumor spheres (with a diameter > 50 μm) were measured by an assessor in a blinded manner. The ratio of sphere formation was calculated as the number of spheres per well divided by the number of seed cells per well. The spheres were dissociated into single cells with trypsin–EDTA for cell passage and subsequent analysis.

For the in vitro extreme limiting dilution assay, single-cell suspensions of the indicated groups were serially diluted to a decreasing cell density (160, 80, 40, 20, 10, 5, and 1 cell per well) and seeded into 96-well plates with an ultra-low attachment surface (Corning). The cells were then incubated under sphere formation conditions (as described above) for 10 days. The presence of spheres in each well was recorded, and the stem cell frequency was calculated using the ELDA tool (https://bioinf.wehi.edu.au/software/elda) [29].

### Soft agar colony formation assay

The soft agar colony formation assay was performed according to a previously published protocol [30]. In brief, a 1% noble agar solution (UltraPure™ Low Melting Point Agarose, #16520050, Thermo Scientific) was mixed with an equal volume of stem cell medium (as described above), and 1.5 mL of the mixture was layered onto the bottom of each well in 6-well plates. For the top layer, cells from the indicated groups were suspended in stem cell medium at a density of 5000 cells per well. Next, the cell suspensions were mixed with 0.6% agar solution at a 1:1 ratio and added to the top of each well (1.5 mL). After 14 days of incubation, the cells were stained with 0.05% crystal violet (#C0775, Sigma–Aldrich), and the number of colonies was counted by an assessor in a blinded manner.

### Cell proliferation assay

Cell proliferation was assessed by an ethynyl-2- deoxyuridine (EdU) assay, with the Click-iT™ Plus EdU Cell Proliferation Kit and Alexa Fluor 488 dye (#C10637, Invitrogen), following the manufacturer’s instructions. Hoechst 33342 was used as a nuclear dye. The cells were then visualized using fluorescence microscopy (Eclipse C1, Nikon), and the percentage of EdU-positive cells was evaluated using Image-Pro Plus (version 6.0, Media Cybernetics, USA).

### Cell viability and cisplatin sensitivity assay

Cell viability and cisplatin sensitivity were evaluated using the Cell Counting Kit-8 assay (#CK04-11, Dojindo), following the manufacturer’s instructions. Briefly, the indicated cells were seeded into 96-well plates at a density of 2000 cells per well and treated with increasing concentrations of cisplatin (0.5–160 μg/mL) for 48 h. Afterward, 10 mL of CCK-8 solution was added to each well and incubated at 37°C for 2 h. The absorbance at 450 nm was measured using a microplate reader (RT-6100, Rayto), and the half inhibitory concentration (IC_50_) value and a dose–response curve (*y* = Bottom + (Top–Bottom)/(1 + 10 ^(Log IC50−*x*) *HillSlope^)) were calculated and plotted using GraphPad Prism (version 8.3.0).

### Lipid analysis

Samples were extracted using the chloroform/methanol (2:1, v/v) method. Briefly, cell suspensions or tissue homogenates were mixed with 150 μL of extraction buffer and 20 μL of ceramide internal standard solution (2 μM). After sufficient vortex mixing, the organic phase was separated and collected into a fresh tube, dried under a stream of nitrogen gas, and stored at 80°C for liquid chromatography–mass spectrometry (LC–MS) analysis. Lipids were measured using a high-performance liquid chromatography system (CSH C18 column, 1.7 μm, 2.1 mm × 100 mm, Waters) and mass spectrometer (Q ExactiveTM Plus Hybrid Quadrupole-Orbitrap, Thermo Scientific). Mass spectra were acquired by Q-Exactive Plus in positive and negative modes. ESI parameters were optimized and preset for all measurements as follows: source temperature, 300 °C; capillary temperature, 350 °C, the ion spray voltage was set at 3000 V, the S-Lens RF level was set at 50%; and the scan range of the instrument was set at m/z 200–1800.

### Immunofluorescent staining

For immunofluorescence staining, cultured cells undergoing the indicated treatments were fixed with 4% paraformaldehyde for 30 min, permeabilized with 0.5% Triton X-100 for 20 min, and blocked in 5% goat serum for 60 min. For immunofluorescence staining of tissues, formalin-fixed, paraffin-embedded (FFPE) slides were deparaffinized with xylene, followed by antigen retrieval with citrate buffer (pH 6.0) and blockade with 5% goat serum. The cells or tissues were then incubated with anti-β-catenin antibody (#8480S, CST) at 4 °C overnight and visualized with Daylight 488 anti-rabbit IgG (#5230–0385, KPL) at 37 °C for 60 min, followed by DAPI (#D1306, Invitrogen) staining at room temperature for 5 min. Finally, the slides were photographed under a fluorescence microscope (Eclipse C1, Nikon).

### IHC and TUNEL staining

Protein expression of LASS2 in FFPE sections was detected by immunohistochemistry. Briefly, antigen retrieval was carried out using the heating method with 10 mM sodium citrate buffer (pH 6.0), followed by incubating the sections in 3% hydrogen peroxide for 10 min to inhibit endogenous peroxidase activity. Then, the sections were incubated with an anti-LASS2 antibody (ab279372, Abcam) at 4 °C overnight, followed by incubation with a biotinylated secondary antibody (#31,800, Invitrogen). Negative controls were acquired by omitting the primary antibody. Finally, the sections were visualized using diaminobenzidine chromogen (#AR1021, Boster) and counterstained with hematoxylin (#AR0005, Boster). Positive expression was defined as brown-yellow granules in the cytoplasm or nucleus. The sections were photographed using Pannoramic 1000 (3DHISTECH Ltd., Hungary) and viewed with CaseViewer (version 2.2.1, 3DHISTECH Ltd., Hungary). The expression levels of each section were determined by the histochemistry score (H-Score) and calculated with QuantCenter (version 2.1, 3DHISTECH Ltd., Hungary). H-Score = ∑ (PI × I) = (percentage of cells of weak intensity × 1) + (percentage of cells of moderate intensity × 2) + (percentage of cells of strong intensity × 3). Target region outlining and scoring were performed by an assessor in a blinded manner. The tissue classification was identified by QuantCenter and the epithelium and stromal were analyzed separately.

Apoptotic cells in tumor tissues were detected with a TUNEL In Situ Apoptosis Kit (HRP-DAB Method) (#E-CK-A331, Elabscience) according to the manufacturer’s instructions. Five visual fields were randomly selected under a × 40 magnification (E100, Nikon) to count the positively stained cells by an assessor in a blinded manner. The percentage of TUNEL-positive cells was evaluated using Image-Pro Plus (version 6.0, Media Cybernetics, USA).

### Reverse-transcription PCR (RT–PCR) and PCR array

Total RNA was extracted from cells or tissues using TRIzol reagent (#15596018, Invitrogen) according to the manufacturer’s instructions. RT–PCR was performed using a ViiA 7 Real-Time PCR System (Applied Biosystems, Foster City, CA). The relative mRNA expression was normalized to GAPDH expression and determined using the 2^−ΔΔCt^ method. The primer sequences are listed in Additional file 3. Additionally, we used the RT^2^ Profiler PCR Array (#PAHS-004ZA, Qiagen) which includes a set of 84 genes involved in chemotherapy response and 5 housekeeping genes to analyze the gene expression profiles of BCSCs following the manufacturer’s instructions (Additional file 4).

### Immunoblotting and coimmunoprecipitation

Nuclear and cytoplasmic proteins were extracted separately using NE-PER Nuclear and Cytoplasmic Extraction Reagents (#78833, Thermo Scientific), and total proteins were extracted using RIPA Lysis and Extraction Buffer (#89901, Thermo Scientific). After quantification using a BCA protein assay kit (#G2026, Servicebio), the protein samples were separated by SDS–PAGE and then electrotransferred onto polyvinylidene difluoride membranes. The membranes were washed with TBST and blocked with 10% nonfat milk. The membranes were then incubated with the indicated primary antibodies overnight, washed with TBST, incubated with peroxidase-conjugated secondary antibodies for 2 h, and visualized using the electrochemical luminescence method. The protein level of GAPDH or Lamin B1 was used as an internal control for the cytoplasm or nucleus, respectively. For the protein half-life assay, 20 μg/mL cycloheximide (#C4859, Sigma–Aldrich) was added to the cell medium after gene manipulation. Cell lysates were collected at the indicated time points and subjected to immunoblotting analysis.

For coimmunoprecipitation, 1 mg of cell lysates was incubated with 2 μg of control IgG or anti-β-catenin antibody at 4°C overnight. The lysates were then incubated with 80 μL Protein A/G PLUS Agarose Beads (#IP05, Millipore) at 4°C for 2 h. The beads were washed with IP lysis buffer (#G2038, Servicebio) five times. The precipitates were examined by immunoblotting using the indicated antibodies. The antibodies used are listed in Additional file 3.

### PP2A immunoprecipitation phosphatase assay

The activity of PP2A was determined using the PP2A Immunoprecipitation Phosphatase Assay Kit (#17–313, Millipore). Briefly, total protein was extracted using IP lysis buffer (#G2038, Servicebio). Then, 200 mg of lysates was incubated with 4 mg of anti-PP2A-C subunit antibody and 40 mL of Protein A agarose beads at 4°C for 2 h with constant rocking. After three washes with TBS and one wash with Ser/Thr Assay Buffer, 60 mL of diluted threonine phosphopeptide (K-R-pT-I-R-R) and 20 mL of Ser/Thr Assay Buffer were added (final reaction concentration was 750 μM), and the mixture was incubated for 10 min at 30°C in a shaking incubator. The mixture was then centrifuged, and 25 μL of supernatant was transferred into each well of a 96-well microtiter plate. The reaction was stopped by adding 100 μL of Malachite Green Phosphate Detection Solution and incubated for 15 min at room temperature. The phosphate standard solution was serially diluted, and the absorbance versus phosphate concentration (pmole) was plotted for a phosphate standard curve. Free phosphate concentrations were quantified by reading the absorbance at 650 nm using a microplate reader (RT-6100, Rayto).

### Chromatin immunoprecipitation (ChIP)

ChIP assays were performed to investigate the binding of β-catenin/TCF4 to the ABCC2 or CD44 promoter in BCSCs. Briefly, after gene manipulation, cells (at a density of 2 × 10^6^) in 100-mm dishes were treated with 1% formaldehyde to crosslink proteins to DNA, followed by sonication. Then, 90 µL of the sonicated chromatin was removed as input, and the remaining chromatin was incubated with 1 µg of control IgG or the specific antibodies overnight at 4 °C. After immunoprecipitation using agarose beads, the protein–DNA complexes were captured and the crosslinks were reversed to release the DNA. The human ABCC2 and CD44 promoter fragments were then amplified using RT–PCR. The primer sequences used are listed in Additional file 3.

### Luciferase reporter assays

The promoter sequence of the ABCC2 or CD44 gene was amplified and cloned into the pGL3-Vector (#E1751, Promega) to generate the ABCC2 or CD44-luciferase reporter plasmid. BCSCs were seeded at a density of 1 × 10^5^ per well in 24-well plates 1 day before infection. Co-infection of pCMV6-control or pCMV6-LASS2 with pGL3-control or luciferase reporter plasmid (100 ng) was performed in BCSCs. Then, the cells were treated with Wnt3a (250 ng/mL) or vehicle (PBS). As an internal transfection control, 1 ng of pRL-TK Renilla plasmid (#E2231, Promega) was also co-transfected into BCSCs. Luciferase activity was measured with a Dual-Luciferase Reporter System (#E1910, Promega) at 24 h after transfection.

A Cignal Finder 10-Pathway Reporter Array (#CCA-106L, SABiosciences) was used to identify relevant pathways mediated by the effect of LASS2 in BCSCs. Briefly, 200 ng pCMV6-LASS2 plasmid or control plasmid was diluted with 50 μL Opti-MEM in each well of the Cignal Finder Array plate followed by resuspension of the reporter assay constructs. After a 5-min incubation, 0.6 mL of Attractene was added to each well. After a 20-min incubation for complex formation, 50 μL of the cell suspension (at a density of 8 × 10^4^ in Opti-MEM) was added to each well containing constructs-Attractene complexes. The cells were then incubated at 37 °C for 24 h. Finally, luciferase activity was determined using the Dual-Luciferase Reporter Assay System (#E1910, Promega). The fold change (pCMV6-LASS2 versus control plasmid) in the activity of each signaling pathway was calculated and plotted using GraphPad Prism 9 (version 8.3.0).

For measuring Wnt/β-catenin activation, the TOPFlash reporter (#12456, Addgene), a Wnt/β-catenin pathway-responsive firefly luciferase reporter plasmid, and a mutant FOPFlash reporter (#12457, Addgene) were transfected into the indicated cells, along with Wnt3a treatment for the indicated time. Luciferase activity was determined using the Dual-Luciferase Reporter Assay System (#E1910, Promega), and Wnt/β-catenin activation was measured based on the fold change in TOPFlash luciferase activity versus FOPFlash luciferase activity.

### Cell-derived xenograft (CDX) model and in vivo limiting dilution assay

BCSCs that were transfected with an LASS2 expressing vector or a control vector were mixed with Matrigel matrix (#356235, Corning) and subcutaneously injected (1 × 10^6^ cells per mouse) into 6-week-old BALB/c nude mice. Once the tumors were palpable (approximately 60 mm^3^ in volume), mice were administered cisplatin intraperitoneally (1 or 2 mg/kg) every 3 days, or (and) XAV939 (25 or 50 mg/kg) every 3 days. PBS or DMSO was used as a vehicle. Tumor volume was measured every week and calculated as ½ (length × width^2^). To verify the stemness of BCSCs, an in vivo limiting dilution assay was performed. Briefly, limited numbers of cells (2 × 10^1^, 2 × 10^2^, 2 × 10^3^, 2 × 10^4^) were subcutaneously injected into 6-week-old BALB/c nude mice. Tumor volume was measured every week. After 8 weeks, the mice were euthanized by cervical dislocation, and the tumors were collected. The stem cell frequency was calculated using ELDA tool (https://bioinf.wehi.edu.au/software/elda) [29].

### Patient-derived xenograft (PDX) model and adeno-associated virus (AAV) treatment

Bladder cancer specimens used to construct PDX models were collected from three patients who progressed after cisplatin-based NAC (Additional file 5). Fresh tumor tissues were collected in RPMI-1640 medium containing ampicillin and streptomycin, and then were minced into small pieces (approximately 5 × 5 mm) and subcutaneously implanted into the flanks of 6-week-old BALB/c nude mice. Once the tumor volume reached approximately 100 mm^3^, the mice were randomly divided into five groups: (1) control; (2) cisplatin treatment; (3) cisplatin combined with AAV9-LASS2 treatment; (4) cisplatin combined with XAV939 treatment; (5) cisplatin combined with AAV9-LASS2 and XAV939 treatment. Normal saline or cisplatin (2 mg/kg) was administered intraperitoneally every 3 days [31]. An AAV9 vector carrying LASS2 (NM_022075.5) and EGFP with a CMV promoter (pAAV9-CMV-hCERS2-T2A-EGFP-WPRE) was generated and packaged by VectorBuilder (VB900132-1479fxd, VectorBuilder Inc). An AAV9-EGFP vector that express EGFP alone was used as a negative control. The titer of the virus was 1 × 10^12^ vg/ml. AAV9-LASS2 or AAV9-EGFP was injected intratumorally in a volume of 100 μL (5 × 10^10^ vg/100 μL) twice per week from day 7 post cisplatin treatment until the end point. In addition, vehicle (DMSO) or XAV939 (50 mg/kg) was administered intraperitoneally every 3 days from day 7 post cisplatin treatment until the endpoint [32]. Tumor volume was measured every week and calculated as ½ (length × width^2^). Adverse effects were evaluated by measuring body weight, organ weight, and hematological parameters. Endpoint criteria included mouse death or poor body condition. When the endpoint was reached, PDX tumors were harvested and divided into three fragments for immunofluorescence staining, histopathological examination, and cell isolation.

To determine the cisplatin sensitivity of PDX tumors, tumors were minced into small fragments and digested with 200 U type I collagenase, 0.01% hyaluronidase, and 0.01% DNase I in RPMI 1640 medium. The cells were then filtered through a cell strainer (70 μm), washed, and suspended in RPMI 1640 medium. Cells from each PDX tumor were seeded in 96-well plates (10^4^ cells per well) and treated with different concentrations of cisplatin for 48 h. Cell viability and cisplatin IC_50_ were measured using the Cell Counting Kit-8 assay (as described above). Mice were housed under specific pathogen-free conditions at Kunming Medical University. All animal procedures were performed under a protocol approved by the Animal Experiment Ethical Committee of Kunming Medical University (Ethical number: kmmu20211135).

### Statistics

Data analysis was conducted using GraphPad Prism (version 8.3.0) and the R program (version 4.1.0). Continuous measures were analyzed using Student’s *t* test (two-tailed) for two groups and one-way ANOVA or the Kruskal–Wallis test for multiple groups. Categorical measures were analyzed by the chi-square test. Correlation analysis was performed using Spearman’s test. Survival data were plotted by the Kaplan–Meier curve and analyzed using the Log-rank test. Data are presented as mean ± standard deviation (SD) from at least three independent experiments. A P value less than 0.05 was considered statistically significant.

## Results

### Loss of LASS2 is an indicator of a poor response in bladder cancer patients who receive cisplatin-based chemotherapy

**Fig. 1:**
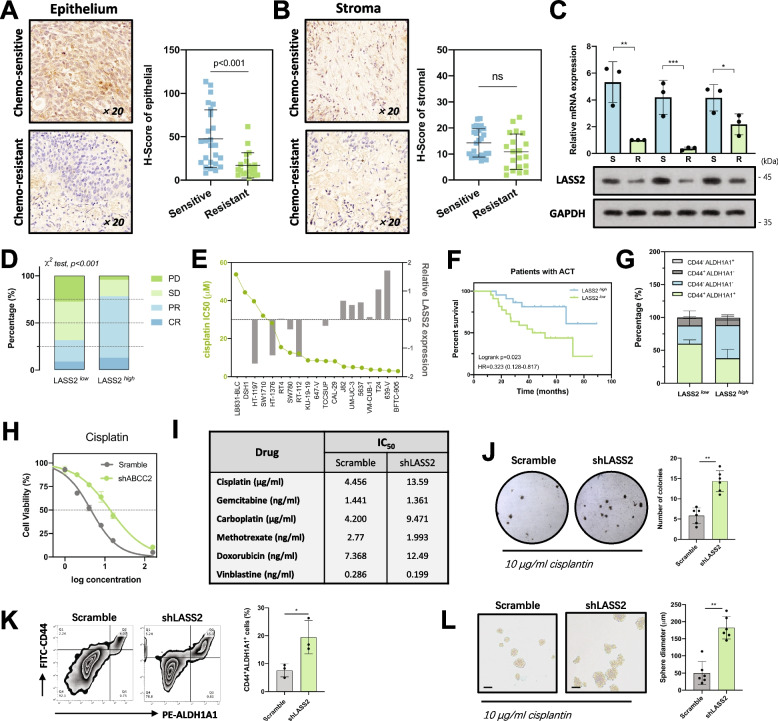
Loss of LASS2 indicates a poor chemotherapy response and confers a stem-like phenotype in bladder cancer.** A, B** Immunohistochemistry staining images and the expression levels of LASS2 (determined by H-Score) in the epithelial (**A**) and stromal (**B**) tissues from chemoresistant and chemosensitive bladder cancer specimens. **C** The mRNA (upper) and protein (lower) expression levels of LASS2 in chemoresistant and chemosensitive bladder cancer specimens. **D** Proportion of different responders to cisplatin-based chemotherapy in our cohort. Data are presented as percentages and analyzed by the chi-square test. **E** Graph showing the LASS2 expression levels and IC_50_ values of the indicated bladder cancer cell lines from the GDSC database. **F** Kaplan–Meier curve and Log-rank test of overall survival for patients received cisplatin-based chemotherapy in our center. **G** Percentage of CD44^+^ALDH1A1^−^, CD44^−^ALDH1A1^+^, CD44^+^ALDH1A1^+^, and CD44^−^ALDH1A1^−^ subpopulations in patients with low expression of LASS2 and those with high expression of LASS2. **H** Cell viability and cisplatin IC_50_ of the indicated cells. **I** Table showing the IC_50_ values of the 5637 cell line treated with different chemotherapy agents. **J** Soft agar colony formation assay of the indicated cells treated with 10 μg/mL cisplatin for 24 h. **K** Flow cytometry analysis and quantitation of the CD44^+^ALDH1A1^+^ subpopulation in LASS2 knockdown and control cells. **L** Sphere formation assay of the indicated cells treated with 10 μg/mL cisplatin for 24 h. Scale bars, 100 μm. Data are presented as the mean ± SD of at least three technical replicates. ns, not significant, *p < 0.05, **p < 0.01, ***p < 0.001, determined by Student’s *t* test and one-way ANOVA

To gain insight into the role of LASS2 in chemoresistance in bladder cancer, paraffin-embedded specimens were analyzed by IHC in a cohort of MIBC patients who underwent cisplatin-based NAC (Additional file 1). According to the immunostaining results, LASS2 was present in the nucleus and cytoplasm of bladder urothelial cells and was almost absent in stromal tissues (Additional file 2: Fig. S1). Chemoresistant tumors (*n* = 20) had significantly lower LASS2 expression than chemosensitive tumors (*n* = 25). This difference was only observed in epithelial tissues but not in stromal tissues (Fig. 1A, B). We further validated these results by performing immunoblotting and qPCR of fresh specimens (Fig. 1C). Importantly, patients with high expression of LASS2 had a better response to cisplatin-based chemotherapy than patients with low expression of LASS2 (Fig. 1D). Furthermore, LASS2 expression levels were negatively associated with cisplatin IC_50_ values based on transcriptional and drug sensitivity data of 19 bladder cancer cell lines from the GDSC database (Fig. 1E). This negative relationship between LASS2 expression levels and cisplatin IC_50_ values of bladder cancer cells was further validated based on our experimental data (Additional file 2: Fig. S3C and D).

We next performed Kaplan–Meier survival analysis to investigate the prognostic role of LASS2. Patients with low expression of LASS2 had similar OS compared to those with high expression (Additional file 6). Notably, when we performed subgroup analysis on patients who underwent cisplatin-based adjuvant chemotherapy, we found that low expression of LASS2 was associated with poorer survival (Fig. 1F). This finding was confirmed using two independent cohorts, GSE70691 and GSE69795 (Additional file 2: Fig. S2A). As LASS2 is involved in ceramide synthesis, we also tested the levels of different ceramide in chemosensitive and chemoresistant specimens. Our results showed that the product of LASS2, C24-ceramide, was lower in chemoresistant specimens compared to chemosensitive specimens (Additional file 2: Fig. S3G). Taken together, these data indicate that LASS2 is downregulated in cisplatin-resistant tumors and that patients with low expression of LASS2 have a poorer response to cisplatin-based chemotherapy.

### Loss of LASS2 confers a stem-like phenotype in bladder cancer

Previous studies have suggested that gene signatures related to stemness are enriched in basal-like bladder cancer [33]. Analysis of this subtype in the TCGA also supports this conclusion, as the basal/squamous subtype demonstrates a more stem-like phenotype [34]. Therefore, we examined the expression of LASS2 and stemness-related genes in different bladder cancer subtypes. Our findings showed that the basal/squamous subtype had low expression of LASS2 and high expression of stemness-related markers, including CD44, CD47, STAT3, and ALDH1A1 (Additional file 2: Fig. S2B). In addition, mesenchymal stem-like breast cancer, which exhibits characteristics of breast cancer stem cells [35, 36], also exhibited low expression of LASS2 (Additional file 2: Fig. S2C and D). Furthermore, bladder cancer tissues with low LASS2 expression had a higher proportion of stem-like cells (CD44^+^ALDH1A1^+^) (Fig. 1G). These results suggest a negative role of LASS2 in regulating stem-like properties. To further confirm our findings, we knocked down the endogenous expression of LASS2 in pBCs (derived from a chemotherapy-naïve patient) via an shRNA knockdown approach, resulting in a significantly increase in cisplatin IC_50_ values (Fig. 1H). Similar results were also observed in cell lines from different cancer types (Additional file 2: Fig. S4A and B). Additionally, LASS2 knockdown enhanced resistance to cisplatin and carboplatin, but not to other commonly used chemotherapy agents in bladder cancer, such as gemcitabine, methotrexate, and vinblastine, indicating a specific effect of LASS2 on platinum (Fig. 1I, Additional file 2: Fig. S4C). Furthermore, LASS2-knockdown pBCs formed more colonies when exposed to cisplatin (Fig. 1J) and led to an expansion of CD44^+^ALDH1A1^+^ subpopulation (Fig. 1K), also enhancing the capability of tumor sphere formation (Fig. 1L). Taken together, our findings suggest that loss of LASS2 may maintain a stem-like phenotype and contribute to cisplatin resistance.

### LASS2 inhibits the stemness and cisplatin resistance of BCSCs

**Fig. 2:**
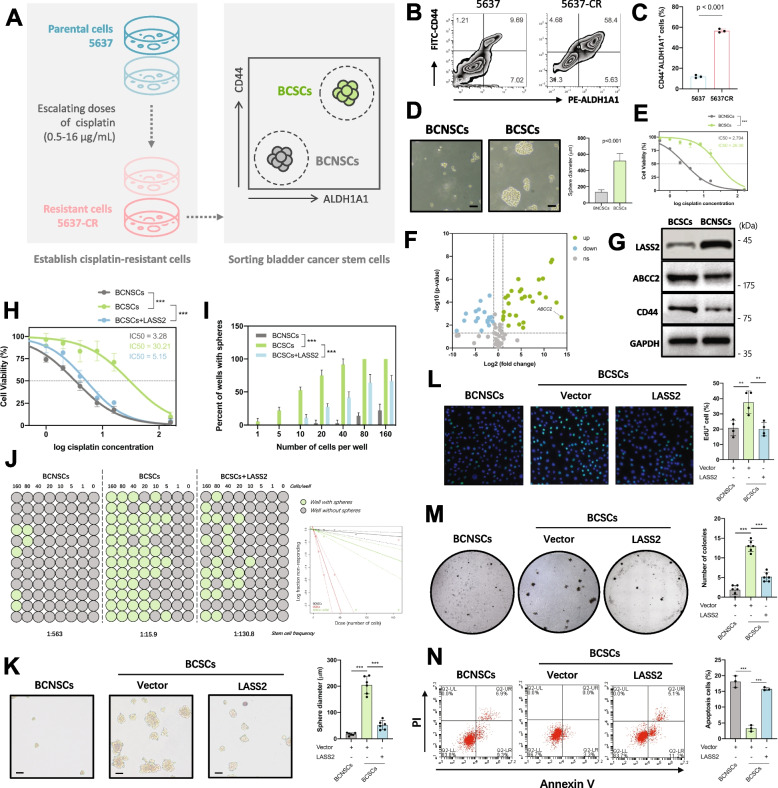
LASS2 inhibits the stemness and cisplatin resistance of BCSCs. **A** A diagram showing the establishment of cisplatin-resistant cells and BCSC sorting. **B, C** Flow cytometry analysis and quantitation of the CD44^+^ALDH1A1^+^ subpopulation in 5637 and 5637-CR cells. **D** Sphere formation assay of BCSCs and BCNSCs. Left: representative images of spheres. Right: comparison of the diameter of spheres. **E** Cell viability and IC_50_ assay of BCSCs and BCNSCs.** F** Differentially expressed resistance-related genes in BCSCs were identified by an RT2 Profiler PCR Array (*p* < 0.05 and fold change > 5 genes were selected). **G** Immunoblotting determining the protein expression levels of LASS2, ABCC2, and CD44 in BCSCs and BCNSCs. **H** Cell viability and IC_50_ assay for the indicated cells treated with 10 μg/mL cisplatin for 24 h. **I** In vitro extreme limiting dilution assay for the indicated cells treated with 10 μg/mL cisplatin for 24 h. **J** Sphere formation frequency of the indicated cells. ELDA software was used to calculate stem cell frequency (left panel) and perform the goodness-of-fit test (right panel). **K–N** Representative images (left panel) and quantification (right panel) of tumor sphere formation (**K**), EdU (**L**), soft agar colony formation (**M**), and apoptosis (**N**) assays for the indicated cells transfected with control or LASS2-overexpressing plasmid and treated with 10 μg/mL cisplatin for 24 h. Scale bars, 100 μm for the tumor sphere formation assay, and 50 μm for the EdU assay. Data are presented as the means ± SD of at least three technical replicates. **p < 0.01, ***p < 0.001, determined by one-way ANOVA

To confirm whether LASS2 plays important role in the stem-like phenotype of bladder cancer, we sorted CD44^+^ALDH1A1^+^ subpopulation from the 5637 cell line (Fig. 2A), which has a predominant enrichment of the basal-like signature [26] (Additional file 2: Fig. S3A), displays a more stem-like phenotype [33], has a higher expression level of CD44 (Additional file 2: Fig. S3B), as well as contains a larger CD44^+^ALDH1A1^+^ subpopulation (Additional file 2: Fig. S3F). Previous studies have demonstrated that cancer stem cells are enriched in chemoresistant cells [37–39]. Consistently, cisplatin-resistant subline of 5637 (5637-CR) were enriched with CD44^+^ALDH1A1^+^ subpopulation compared to parental cells (Fig. 2B, C). Keeping with these findings, we sorted 5637-CR cells into a CD44^+^ALDH1A1^+^ subpopulation and a CD44^−^ALDH1A1^−^ subpopulation (Additional file 2: Fig. S5A and B). The CD44^+^ALDH1A1^+^ subpopulation, which is referred to as bladder cancer stem cells (BCSCs), displayed enhanced tumor sphere formation and resistance to cisplatin. The CD44^−^ALDH1A1^−^ subpopulation, which is referred to as bladder cancer non-stem cells (BCNSCs), displayed impaired tumor sphere formation and was sensitive to cisplatin (Fig. 2D, E). BCSCs also expressed higher levels of CSC markers and had a remarkably stronger tumorigenic capacity and a higher tumorigenic cell frequency compared to BCNSC (Additional file 2: Fig. S5C-H). These findings are consistent with a previous study that demonstrated that CD44^+^ALDH^+^ distinguishes bladder cancer stem cell sublines [40].

To better understand the characteristics of BCSCs and explore the mechanisms of cisplatin resistance, we evaluated 84 drug resistance-related genes using an RT^2^ Profiler PCR Array (Additional file 4). Distinct expression patterns of drug resistance-related genes were observed between BCSCs and BCNSCs (Additional file 2: Fig. S5J). We identified 21 drug resistance-related genes that were upregulated (fold change > 5 and *p* < 0.05) in BCSCs, with ABCC2 being the most highly upregulated gene (Fig. 2F). ABCC2 is a major ATP-binding cassette transporter responsible for increasing the efflux of cisplatin [41] and has been associated with cisplatin resistance in various cancers, including bladder cancer [42–44]. Immunoblotting analysis showed that BSCSs had lower expression of LASS2 and higher expression of ABCC2 and CD44 (Fig. 2G). In addition, BCSCs had lower levels of C22- and C24-ceramide (Additional file 2: Fig. S5I), which are products of LASS2 [45].

To investigate whether LASS2 expression is related to cisplatin resistance in BCSCs, we overexpressed LASS2 in BCSCs by transfecting the pCMV6-LASS2 expression plasmid. We found that LASS2 overexpression significantly decreased the viability and IC_50_ values of cisplatin in BCSCs (Fig. 2H). EdU assay showed that LASS2 overexpression significantly decreased the proliferation ability of BCSCs (Fig. 2L). Soft agar colony formation assay showed that BCSCs generated larger and more colonies than BCNSCs under exposure to cisplatin, while LASS2 overexpression impaired the anchorage-independent growth ability of BCSCs (Fig. 2M). LASS2 overexpression also increased cisplatin-induced cell apoptosis (Fig. 2N). Furthermore, tumor sphere formation and in vitro extreme limiting dilution assays showed that LASS2 overexpression reduced both the number and size of spheres (Fig. 2K) and the sphere formation frequency (Fig. 2I, J). These results suggest that LASS2 decreases the self-renewal ability of BCSCs and increases their sensitivity to cisplatin.

### LASS2 attenuates Wnt/β-catenin signaling by inhibiting β-catenin nuclear translocation

**Fig. 3:**
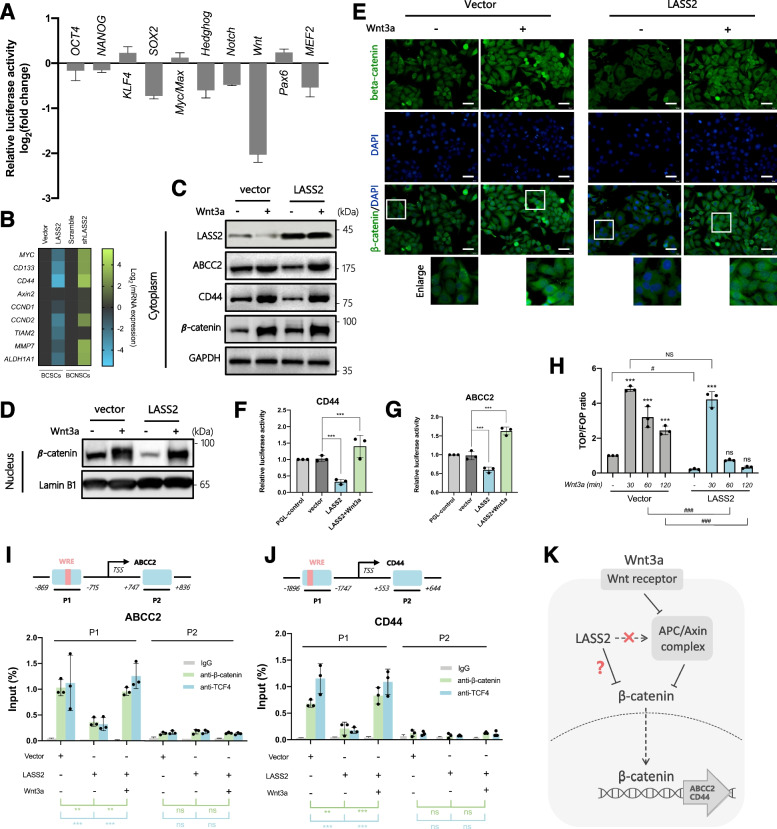
LASS2 attenuates Wnt/β-catenin signaling by inhibiting β-catenin nuclear translocation.** A** Screening of differentially activated pathways in LASS2-overexpressing BCSCs versus control BCSCs by Cignal Finder 10-Pathway Reporter Array. **B** Heatmap showing the expression pattern of Wnt/β-catenin downstream genes in BCSCs transfected with the indicated plasmids. **C, D** Immunoblotting of the indicated cytoplasmic (**C**) or nuclear (**D**) proteins in the control or LASS2-overexpressing BCSCs treated with vehicle (PBS) or 250 ng/mL Wnt3a for 6 h. **E** Immunofluorescent staining images of the indicated cells treated with either Wnt3a (250 ng/mL) or vehicle (PBS) for 6 h. Scale bar, 20 μm. **F, G** Luciferase activity of the ABCC2-reporter plasmid in BCSCs transfected with the indicated plasmids and treated with or without Wnt3a (250 ng/mL) for 30 min. Data are presented as the means ± SD of three technical replicates. ***p < 0.001, determined by one-way ANOVA. **H** TOPFlash/FOPFlash reporter gene assay in control and LASS2-overexpressing BCSCs treated with Wnt3a (250 ng/mL) or vehicle (PBS) for the indicated periods. Data are presented as the means ± SD of three technical replicates. ***p < 0.001 versus vehicle, ns, not significant versus vehicle; #*p* < 0.05, ###*p* < 0.001, NS, not significant, determined by one-way ANOVA. **I, J** Upper panel: schematic illustrations of the primer fragments of the ABCC2 (**I**) and CD44 (**J**) promoters. Lower panel: control and LASS2-overexpressing BCSCs treated with or without Wnt3a (250 ng/ml) for 30 min were subjected to chromatin immunoprecipitation using antibodies against β-catenin and TCF4, followed by RT–PCR for ABCC2 (**I**) and CD44 (**J**) gene fragments. Data are presented as the percentage of input. Data are presented as the means ± SD of three technical replicates. ns, not significant, **p < 0.01, ***p < 0.001, determined by one-way ANOVA. **K** A diagram illustrating the possible mechanisms of LASS2 in regulating Wnt/β-catenin signaling

We next screened stemness-related pathways mediated by LASS2 in BCSCs using the Cignal Finder 10-Pathways Reporter Array, which contains stem cell-focused reporters that enable us to measure the activities of downstream transcription factors. LASS2 overexpression decreased the transcriptional activity of most stemness-related pathways in BCSCs, with the most dramatically decreased pathway being the Wnt signaling pathway (Fig. 3A, Additional file 7). Consistent with these results, pathway activity analysis based on RPPA data showed that LASS2-high tumors had lower activity of EMT-related and Wnt pathways than LASS2-low tumors in multiple cancers, including bladder cancer, breast cancer, lung cancer, stomach cancer, and testicular cancer (Additional file 2: Fig. S3H, Additional file 8). Additionally, LASS2 overexpression decreased, while LASS2 knockdown increased, the mRNA expression of multiple Wnt/β-catenin downstream genes (Fig. 3B). These results indicated that LASS2 might negatively regulate Wnt signaling. Immunoblotting analysis showed that LASS2 overexpression markedly decreased the protein expression of CD44 and ABCC2 in the absence of Wnt3a, but not in the presence of Wnt3a (Fig. 3C). CD44 is a typical CSC marker and ABCC2 may mediate cisplatin resistance in BCSCs (Fig. 2F). Furthermore, the promoters of CD44 and ABCC2 contain a Wnt-responsive element (WRE), which are the binding sites for β-catenin/TCF4 (Fig. 3I, J).

As β-catenin is a crucial downstream component of the canonical Wnt signaling pathway, we measured the protein levels of β-catenin in total cell lysates and nuclear lysates of BCSCs. We found that overexpression of LASS2 did not significantly alter the protein level of β-catenin in total cell lysates, but significantly decreased the protein level of β-catenin in the nucleus (Fig. 3C, D). These results suggested that LASS2 might inhibit the redistribution of cytoplasmic β-catenin to nuclear localization. We further confirmed this conclusion through immunofluorescence assays (Fig. 3E). The APC/Axin complex inhibits the nuclear translocation of β-catenin in a canonical Wnt/β-catenin pathway [46]. Therefore, we considered whether LASS2 functions by regulating the APC/Axin complex. To address this possibility, we used Wnt3a to activate the Fz/LRP receptor, which subsequently led to blockade of the APC/Axin complex and nuclear translocation of β-catenin. We found that overexpression of LASS2 did not inhibit the Wnt3a-induced nuclear translocation of β-catenin (Fig. 3D, E), suggesting that LASS2 might not be involved in the regulation of the APC/Axin complex.

After translocation to the nucleus, nuclear β-catenin then binds to TCF/LEF transcription factors, leading to the transcription and expression of Wnt-responsive genes. Therefore, we examined whether LASS2 inhibited β-catenin/TCF-mediated transcriptional activity. The TOPflash and FOPflash luciferase reporter assays showed that LASS2 overexpression inhibited the activity of β-catenin/TCF reporter in the absence of Wnt3a, but not in the presence of Wnt3a. After Wnt3a stimulation, the activity of β-catenin/TCF reporter declined more quickly to the initial level in LASS2-overexpressing cells than in control cells (Fig. 3H). Furthermore, luciferase reporter assays showed that LASS2 overexpression decreased the luciferase activity driven by the promoters of ABCC2 and CD44 in BCSCs, but not in the treatment of Wnt3a (Fig. 3F, G). Additionally, ChIP assays confirmed that LASS2 overexpression hindered the occupation of β-catenin/TCF4 on the promotors of ABCC2 and CD44. However, the inhibitory role of LASS2 was weakened when Wnt signaling was activated by Wnt3a (Fig. 3I, J). In summary, these findings indicate that LASS2 decreases the transcriptional activity of β-catenin/TCF4 by preventing the translocation of β-catenin to the nucleus, independently of the APC/Axin complex (Fig. 3K).

### PP2A inactivation is necessary for LASS2-induced accumulation of phospho-β-catenin

**Fig. 4:**
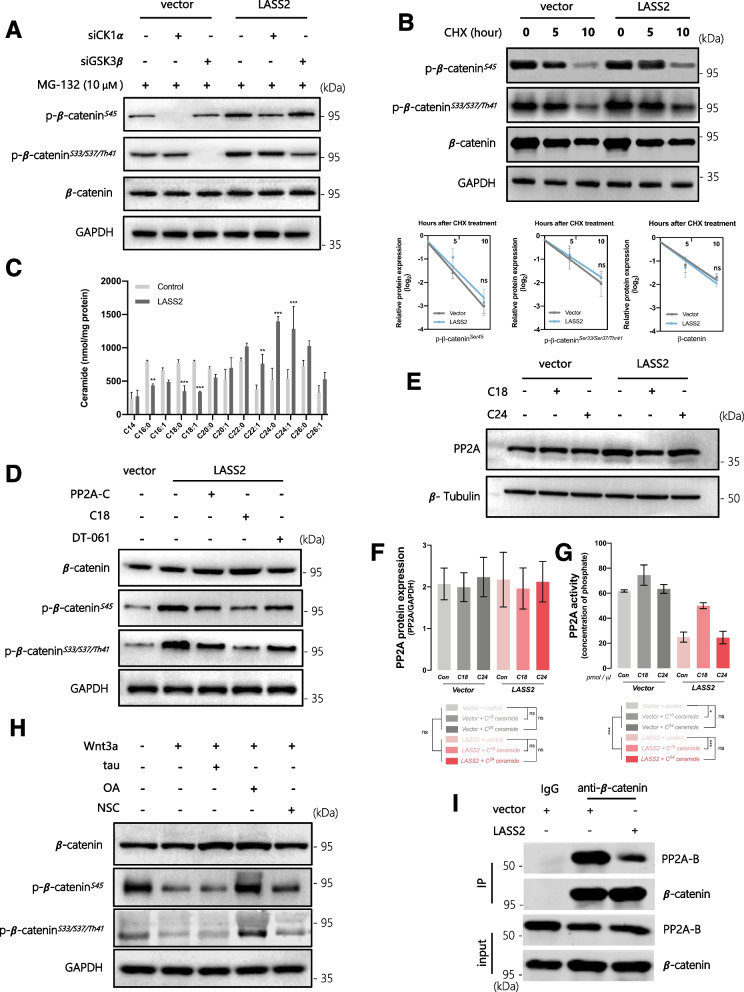
LASS2 suppresses the dephosphorylation of β-catenin by inhibiting PP2A activity and dissociating PP2A from β-catenin. **A** LASS2-overexpressing or control BCSCs were transfected with the indicated siRNAs, followed by treatment with MG-132 (10 μM) for 6 h. Cell lysates were harvested for immunoblotting assays. **B** LASS2-overexpressing or control BCSCs were treated with 20 μg/mL cycloheximide (CHX) for the indicated periods, followed by immunoblotting. The band density was normalized to the 0-time point. **C** Quantitative analysis of ceramides by LC–MS in BCSCs transfected with LASS2-overexpressing or control plasmids. **D** PP2A was activated in LASS2-overexpressing BCSCs by transfection with a PP2A-C subunit plasmid, and treatment with C18-ceramide (100 μM) or DT-061(10 μM) for 6 h. Cell lysates were harvested for immunoblotting assays. **E–G** LASS2-overexpressing or control BCSCs were treated with 100 μM C18- or C24-ceramide for 6 h. Cell lysates were harvested for immunoblotting assays (**E, F**) and PP2A immunoprecipitation phosphatase assays (**G**). **H** BCSCs were pretreated with MG-132 (10 μM) for 6 h, followed by treatment with Wnt3a (250 ng/mL), tautomycetin (1 nM), okadaic acid (0.1 nM), or NSC117079 (50 μM) for additional 2 h. PBS was used as a control. Cell lysates were harvested for immunoblotting assays. **I** Coimmunoprecipitation of β-catenin and the PP2A-B subunit in BCSCs with or without LASS2 overexpression. Data are presented as the mean ± SD of three independent experiments. ns, not significant, *p < 0.05, **p < 0.01, ***p < 0.001, determined by Student’s *t* test or one-way ANOVA

The phosphorylation of cytosolic β-catenin is crucial for its distribution and functions. The non-phosphorylated form of β-catenin can move freely to the nucleus and activate target genes. In contrast, the phosphorylated form of β-catenin is ubiquitinated and targeted for rapid degradation by the proteasome [47]. In the canonical WNT/β-catenin pathway, the APC/Axin complex interacts with GSK3β, CK1α, and β-catenin to coordinate the sequential phosphorylation of β-catenin at Ser45 by CK1 and then at Ser33/37/Thr41 by GSK3β [48]. We examined the phosphorylation status of β-catenin in BCSCs, but analyzing β-catenin phosphorylation was difficult due to the rapid degradation of phospho-β-catenin. Thus, we used a proteasome inhibitor, MG132, to inhibit the degradation of phospho-β-catenin. After blocking APC/Axin complex-mediated phosphorylation with siCK1α or siGSK3β, phosphorylated β-catenin-Ser45 and phosphorylated β-catenin-Ser33/37/Thr41 were significantly decreased, respectively. However, the inhibition of β-catenin phosphorylation by siCK1α or siGSK3β was reversed by LASS2 overexpression. Additionally, LASS2 overexpression did not affect the total β-catenin (Fig. 4A). Because the signaling of LASS2 is independent of APC/Axin complex-mediated phosphorylation, we speculated that the accumulation of phospho-β-catenin induced by LASS2 overexpression might be attributed to the inhibition of degradation or dephosphorylation. To further test whether LASS2 regulates the degradation of β-catenin, we determined the protein half-life of β-catenin using cycloheximide (CHX) to block new protein synthesis. We found that LASS2 overexpression in BCSCs did not affect the protein half-lives of either phospho-β-catenin or total β-catenin (Fig. 4B). According to these results, we suspected that LASS2 might block the dephosphorylation of β-catenin, leading to the accumulation of phospho-β-catenin.

It has been reported that the dephosphorylation of β-catenin is mediated by PP2A, a serine/threonine phosphatase that positively regulates β-catenin signaling [49, 50]. Therefore, we investigated whether PP2A is involved in the LASS2-induced accumulation of phospho-β-catenin. The activation of PP2A by transfection with a PP2A catalytic subunit (PP2A-C) expression plasmid in BCSCs inhibited LASS2-induced accumulation of phospho-β-catenin (Fig. 4D), suggesting that LASS2 might inhibit β-catenin dephosphorylation by regulating PP2A. Next, we found that LASS2 overexpression or ceramide stimulation did not affect the protein expression level of PP2A (Fig. 4E, F), while the in vitro phosphatase assay showed that LASS2 overexpression significantly inhibited the activity of PP2A (Fig. 4G). Additionally, LASS2 functions as a ceramide synthetase, and its overexpression decreases the level of C18-ceramide and increases the level of C24-ceramide [51], and similar results were observed in BCSCs (Fig. 4C). Notably, C18-ceramide was higher in chemoresistant bladder cancer (Additional file 2: Fig. S3G), and it has been reported to bind to inhibitor 2 of PP2A (I2PP2A), inhibiting its activity and thereby activating PP2A [52, 53]. These results help to explain how LASS2 inhibits PP2A activity by regulating ceramide metabolism. Indeed, treatment with exogenous C18-ceramide, but not C24-ceramide, significantly reversed the inhibitory effect of LASS2 on PP2A activity (Fig. 4E). Furthermore, the activation of PP2A by exogenous C18-ceramide or a specific activator (DT-061) in BCSCs reversed the enhancement of β-catenin phosphorylation caused by LASS2 overexpression (Fig. 4D). These results indicate that the inactivation of PP2A is necessary for the LASS2-induced increase in phospho-β-catenin.

It has been reported that some ceramide-activated phosphatases other than PP2A, such as PHLPP1 [54] and PP1 [55], may mediate the dephosphorylation of β-catenin. Therefore, we tested the dephosphorylation effect of several phosphatases on β-catenin in BCSCs. Inhibitors of PP1 (tautomycetin) and PHLPP1 (NSC117079) failed to suppress the dephosphorylation effect on β-catenin (Fig. 4H), suggesting that PP1 and PHLPP1 might not be involved in the regulation of β-catenin phosphorylation. In line with previous studies [56, 57], okadaic acid, a high-affinity inhibitor of PP2A, effectively suppressed the dephosphorylation effect on β-catenin (Fig. 4H). Furthermore, we conducted a coimmunoprecipitation assay to determine whether the interaction between PP2A and β-catenin is affected by LASS2. The results showed that LASS2 overexpression disrupted the co-precipitation of the β-catenin and PP2A-B subunit (Fig. 4I), which have previously been reported to coimmunoprecipitate with β-catenin [50, 56, 58]. Overall, these results suggest that LASS2 inhibits the dephosphorylation of β-catenin by inhibiting PP2A activity and dissociating PP2A from β-catenin.

### Targeting LASS2 sensitizes bladder cancer to cisplatin treatment in vitro and in vivo

**Fig. 5:**
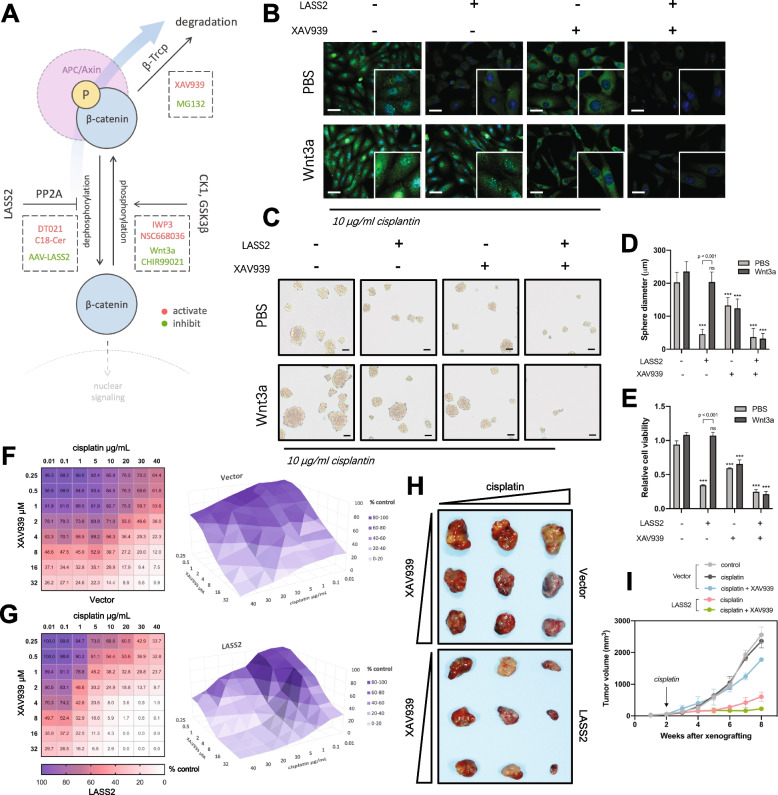
Therapeutic strategies sensitizing bladder cancer to cisplatin treatment by targeting LASS2 and β-catenin signaling.** A** A diagram illustrating the anticancer therapies targeting β-catenin signaling. The blue arrow indicates the treatment strategy by promoting phosphorylation, promoting degradation, and inhibiting dephosphorylation of β-catenin. **B** Immunofluorescent staining of β-catenin in BCSCs with the indicated treatments. Scale bars, 10 μm. **C, D** Representative images (**C**) and quantification (**D**) of the tumor sphere formation assay for BCSCs with the indicated treatments. Scale bars, 100 μm. **E** Cell viability assay of BCSCs with the indicated treatments. Data are presented as the mean ± SD of three independent experiments, ***p < 0.001, determined by Student’s t test or one-way ANOVA. **F, G** Growth effects of combining cisplatin and XAV939 in control (**F**) or LASS2-overexpressing (**G**) BCSCs. The dose matrix in which percentages of growth inhibition relative to untreated control are indicated in each grid and visualized using a color scale. **H** Images of subcutaneous tumors formed by BCSCs with the indicated treatments.** I** Tumor growth curves of CDX models with the indicated treatments

The balance of phosphorylation and dephosphorylation maintains the stability of β-catenin and its signals. Dephosphorylation allows free β-catenin to translocate to the nucleus, whereas phosphorylation of β-catenin creates a binding site for the E3 ubiquitin ligase β-Trcp, leading to β-catenin ubiquitination and degradation [59]. Our findings reveal that overexpression of LASS2 inhibits dephosphorylation, subsequently increasing phospho-β-catenin and attenuating β-catenin signaling, implicating a possible anticancer strategy. However, the APC/Axin complex becomes saturated due to the increasing phospho-β-catenin, leading newly synthesized β-catenin to accumulate and translocate to the nucleus to activate target genes. Therefore, we speculated that a combination of AAV-LASS2 (inhibiting dephosphorylation) and XAV939 (a tankyrase inhibitor that promotes β-catenin degradation) would be a promising approach for blocking β-catenin signaling and overcoming cisplatin resistance (Fig. 5A).

The tumor sphere formation assay and cell viability assay showed that LASS2 overexpression effectively sensitizes BCSCs to cisplatin, but it was not fully effective in BCSCs treated with Wnt3a. XAV939 also increased cisplatin sensitivity partially, but it had a weaker effect than LASS2. The combination of LASS2 overexpression and XAV939 significantly increased cisplatin sensitivity in both BCSCs treated with or without Wnt3a (Fig. 5C–E). Furthermore, immunofluorescent staining revealed that LASS2 overexpression significantly decreased nuclear β-catenin, which was not altered in cells treated with Wnt3a, further supporting that β-catenin nuclear translocation conferred by LASS2 is not dependent on APC/Axin complex-mediated phosphorylation. The combination of LASS2 overexpression and XAV939 significantly decreased not only nuclear β-catenin but also total β-catenin (Fig. 5B). These data suggest that the combination of LASS2 overexpression and XAV939 resulted in cisplatin sensitization and inhibition of tumor growth, even when Wnt signaling was activated.

We next evaluated the synergistic efficacy of LASS2 and XAV939 in abrogating cisplatin resistance in vitro and in vivo. Our results showed that cisplatin and XAV939 synergistically inhibited cell growth with a combination index of 0.43 (Fig. 5F). In LASS2-overexpressing BCSCs, cisplatin in combination with XAV939 resulted in an enhanced synergistic anticancer effect with a combination index of 0.25 (Fig. 5G). We also confirmed these results in a BCSC-derived xenograft model, in which LASS2 and its combination with XAV939 significantly increased the anti-tumor effect of cisplatin (Fig. 5H, I).

**Fig. 6:**
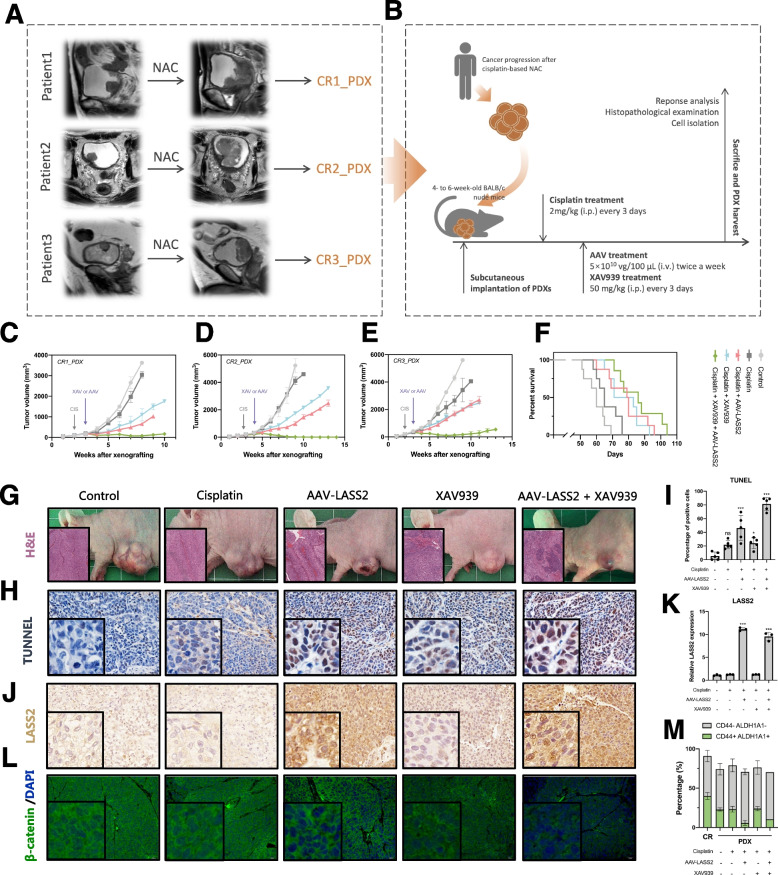
A combination of AAV-LASS2 and XAV939 overcomes cisplatin resistance in bladder cancer.** A** Selected chemoresistant patients for establishing PDX models. **B** A diagram illustrating the experiments of the PDX models. **C–E** Tumor growth curves of PDX tumors with the indicated treatments. **F** Survival rates of mice with the indicated treatments. **G** Representative images and H&E staining of PDX tumors with the indicated treatments. **H, I** Apoptosis assay by TUNEL staining of the indicated PDX tumors. **J, K** Immunohistochemistry staining of LASS2 in the indicated PDX tumors. **L** Immunofluorescent staining of β-catenin in the indicated PDX tumors. **M** Percentage of the CD44^+^ALDH1A1^+^ subpopulation in the indicated PDX tumors

We then established cisplatin-resistant PDX models using bladder tumors isolated from patients who progressed after cisplatin-based NAC (Fig. 6A, B). Our results showed that overexpression of LASS2 by an AAV approach in combination with XAV939 effectively increased cisplatin sensitivity and reduced PDX tumor growth compared with a single treatment (Fig. 6C–G). AAV-LASS2 treatment significantly increased the sensitivity of cisplatin (Fig. 6G, Additional file 2: Fig. S6A), induced apoptotic cell death (Fig. 6H, I), upregulated the expression of LASS2 (Fig. 6J, K), and decreased nuclear β-catenin and the expression of CD44 in PDX tumors (Fig. 6L, Additional file 2: Fig. S6C). Treatment with XAV939 decreased total β-catenin but not nuclear β-catenin (Fig. 6L). Furthermore, AAV-LASS2 treatment reduced the percentage of the CD44^+^ALDH1A1^+^ subpopulation in PDX tumors (Fig. 6M, Additional file 2: Fig. S6B). Additionally, treatment with AAV-LASS2 or XAV939 did not cause any significant changes in body weight, organ weight, or hematopoietic properties (Additional file 2: Fig. S7). These findings together suggest that LASS2 plays a role in inhibiting β-catenin signaling and that a combination of AAV-LASS2 and XAV939 is a promising strategy for overcoming cisplatin resistance in bladder cancer.

## Discussion

Neoadjuvant and adjuvant chemotherapy are important components of standard care for bladder cancer, but the benefits of cisplatin-based chemotherapy are limited to a subset of patients. A recent clinical trial (NCT01812369) found that only approximately 39% of patients had a pathological response to neoadjuvant cisplatin-based chemotherapy (ddMVAC or GC procedure), while approximate 42% of patients experienced progression [60]. Thereafter, cisplatin resistance remains a major challenge in the treatment of bladder cancer. Xie et al. found NAT10 overexpression was associated with chemoresistance, recurrence, and worse clinical outcome in patients with bladder cancer [61]. Recent molecular classification studies have advanced our understanding of the biology of bladder cancer [24]. Sjödahl et al. [62] and Taber et al. [63] found worse pathological and prognostic outcomes in basal/squamous subtypes following neoadjuvant chemotherapy. Robertson et al. [34] showed that the basal/squamous subtype was characterized by stemness-related genes, which are involved in platinum resistance [64]. We also found that the basal/squamous subtype was characterized by high expression of stemness-related genes and low expression of LASS2, indicating associations between LASS2, stemness, and chemotherapy resistance. Indeed, we confirmed that low expression of LASS2 indicated a poor response to cisplatin-based chemotherapy. Although the expression of LASS2 was not associated with the prognosis of total bladder cancer patients, we found that patients with low expression of LASS2 had a worse prognosis in a subgroup analysis of those receiving cisplatin-based chemotherapy. Moreover, we found that the downregulation of LASS2 was associated with an increase in BCSCs proportion and contributed to cisplatin resistance and stemness features in vitro. Thus, these results suggest a link between LASS2 and the chemoresistance of cancer stem cells and encourage us to explore the underlying mechanisms.

LASS2, also known as ceramide synthase 2 (CERS2), is involved in the synthesis of very-long-chain ceramides (chain length C22-C27) by transferring an acyl chain from acyl-CoA to a sphingoid base [11]. In various cancers, LASS2 has been shown to negatively regulate tumorigenesis and metastasis, including bladder cancer [21], prostate cancer [65], breast cancer [66], hepatocellular carcinoma [18], and thyroid cancer [67]. We previously identified LASS2 as a tumor suppressor gene in bladder cancer [68]. However, the functions of LASS2 and the underlying mechanisms have not been fully clarified. This study found that overexpression of LASS2 increased sensitivity to cisplatin and inhibited the stem cell-like phenotype through the Wnt/β-catenin pathway. LASS2 overexpression led to an accumulation of phospho-β-catenin in the cytoplasm, resulting in a decrease in nuclear β-catenin. The nuclear distribution of β-catenin is important for its downstream signaling, which leads to enhanced chemoresistance in cancers [69–72]. We have previously tried knockdown of LASS2 in BCNSCs. BCNSCs grow slowly, and it cannot form cell spheres in an ultra-low attachment surface (Corning). We have tried several times to transfect lentivirus or plasmids into BCNSCs, but the cells died within 1 or 2 days after each transfection experiment. At present, the cause of BCNSCs’ death is unknown. We simply speculate that BCNSCs are vulnerable to external stimuli due to stem cell culture condition, and we will continue to explore what causes BCNSCs’ death. In addition, we have also tried to detect the changes of stemness characteristics after knockdown of LASS2 in BCSCs. Because LASS2 is expressed at a low level in BCSCs, there was no significant difference between control and shLASS2 groups in BCSCs. In agreement with our results, a recent study [65] reported that LASS2 overexpression increased β-catenin phosphorylation and inhibited prostate carcinogenesis. They also concluded that LASS2 promoted β‐catenin degradation, but this result was not very credible because they did not block protein synthesis during the analysis of protein degradation.

**Fig. 7:**
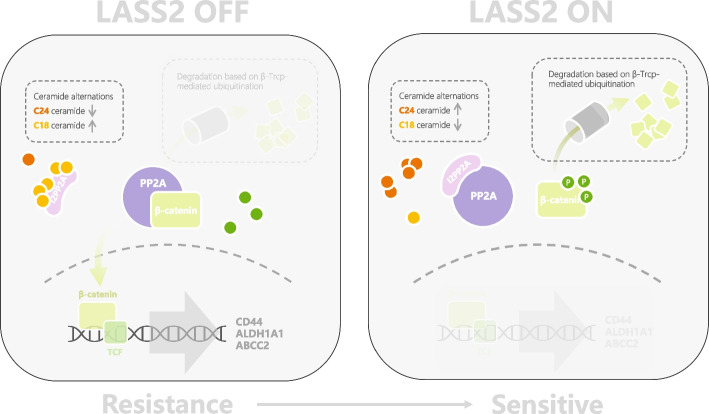
Proposed model for the role of LASS2 in cisplatin resistance in bladder cancer. Left: Loss of LASS2 leads to activation of PP2A by increasing C18-ceramide, while PP2A dephosphorylates β-catenin to promote nuclear translocation and activate β-catenin/TCF4 signaling. Right: LASS2 decreases PP2A activity by reducing C18-ceramide and dissociating PP2A from β-catenin, leading to the degradation of phospho-β-catenin

There are three explanations for the accumulation of phospho-β-catenin: activation of phosphorylation kinases (such as GSK3β or CK1α), inhibition of ubiquitin–proteasome-mediated degradation of phospho-β-catenin, or inhibition of dephosphorylation of phospho-β-catenin. Here, we found that LASS2 did not inhibit the Wnt3a-induced nuclear translocation of β-catenin and did not affect the protein half-lives of either phospho-β-catenin or total β-catenin. Therefore, we excluded the possibility that LASS2 increases phospho-β-catenin by activating the APC/Axin complex or inhibiting ubiquitin–proteasome-mediated degradation and focused on the role of LASS2 in inhibiting dephosphorylation. Furthermore, our data supported that PP2A was responsible for the LASS2-induced accumulation of phospho-β-catenin. Two mechanisms were identified to explain the associations of LASS2 and PP2A: (1) LASS2 decreases PP2A activity by reducing C18-ceramide levels, leading to impairment of β-catenin dephosphorylation; (2) LASS2 inhibits the interaction of PP2A and β-catenin to disable β-catenin dephosphorylation. Collectively, these findings reveal novel mechanisms in which LASS2 inhibits the dephosphorylation of β-catenin by inhibiting the activity of PP2A and dissociating PP2A from β-catenin (Fig. 7).

To interpret our data regarding LASS2-regulated β-catenin signaling in BCSCs, several precautions should be taken. Firstly, there are six ceramide synthases (CERS1-CERS6) found in humans, and their distribution varies in different tissues [13]. We focused on LASS2 (CERS2) as it is a highly expressed gene across cancers, as well as it has a lower expression level in chemoresistant tumors than chemosensitive tumors. Compared with other ceramide synthases, LASS2 has a significant CNV, leading to gene amplification and mRNA expression in pan-cancer (Additional file 2: Fig. S8). Although these six ceramide synthases have a common role in altering ceramide metabolism, the other five ceramide synthetases do not share the role of LASS2 in cancers, suggesting that other underlying mechanisms in addition to ceramide metabolism, should be considered. Secondly, in this study, the CD44^+^ALDH1A1^+^ subpopulation was referred to as bladder cancer stem-like cells, which is consistent with a previous study [40]. However, different biomarkers (Lineage^−^CD44^+^CK5^+^CK20^−^ [73], BCMab1^+^CD44^+^ [74], CD31^−^CD45^−^CD44^+^ [75], MUC1^−^CD44v6^+^ [76]) have been used to isolate bladder cancer stem cells in different studies. Experiments using different subpopulations derived based on different markers may yield different results, and these effects on the study results cannot be ruled out. Thirdly, we identified ABCC2 as the most upregulated drug resistance gene in BCSCs. However, apart from ABCC2, other downstream genes of β-catenin have been reported to be associated with cisplatin resistance, such as c-Myc [77], CCND2 [78], and CCND1 [79]. In fact, except for ABCC2, we also identified several drug resistance and highly expressed genes in BCSCs, such as BRCA1, GSTP1, and IGF1R. Nevertheless, research on multiple genes may be complicated, and the roles of these genes need further exploration.

Wnt/β-catenin signaling plays a critical role in the self-renewal and chemotherapy resistance of cancer stem cells [69–72]. Therefore, targeting Wnt/β-catenin signaling might be a promising therapeutic approach for inhibiting BCSCs. Canonical WNT/β-catenin targeting has long been the focus for anticancer drug development, and several drugs targeting Wnt/β-catenin signaling have entered clinical trials [80]. These drugs mainly promote the phosphorylation of β-catenin, but promoting the degradation of β-catenin is also a promising approach. For example, tankyrase inhibitors (such as XAV939) are potential anticancer drugs that can promote the degradation of β-catenin and significantly inhibit the β-catenin signaling pathway [81–83]. Some drugs targeting tankyrase have shown favorable anticancer results in preclinical tests and have entered phase 2 clinical trials (NCT05475184, NCT03562832). However, drugs such as tankyrase inhibitors that activate ubiquitin–proteasome-mediated degradation may be ineffective if the dephosphorylation process is too strong. If phospho-β-catenin is rapidly dephosphorylated, it cannot be degraded by the ubiquitin–proteasome pathway. Therefore, we speculated that the promotion of degradation combined with overexpression of LASS2, which inhibits dephosphorylation and maintains the phosphorylation of β-catenin, would maximize the blockade of β-catenin signaling. We validated this hypothesis in CDX and PDX mouse models. The results showed that inhibiting dephosphorylation (with AAV-LASS2) or inhibiting degradation (with XAV939) alone showed a modest effect on cisplatin-resistant disease, while the combinatory approach displayed an enhanced effect for the treatment of cisplatin-resistant disease.

## Conclusions

In summary, we have shown that LASS2 loss is more common in patients who are resistant to cisplatin. LASS2 is also lost in a subset of stem-like bladder cancer cells. LASS2 overexpression inhibits the stemness and alleviates cisplatin resistance. Furthermore, we identify dephosphorylation of β-catenin mediated by inactivation of PP2A and dissociation of PP2A-β-catenin as a mechanism of LASS2. Our results provide a rationale for a combinatory dephosphorylation- and degradation-inhibiting strategy to treat cisplatin-resistant bladder cancer. Future pharmacological studies and clinical trials are required to evaluate and optimize this therapeutic strategy.

### Supplementary Information

**Additional file 1.** Patient information.

**Additional file 2:** **Fig. S1.** High-resolution images of immunohistochemical staining. **Fig. S2.** A, Kaplan–Meier curve and Log-rank test of overall survival for patients received cisplatin-based chemotherapy in GSE70691 and GSE69795 datasets. B, The expression levels of LASS2 and stemness-related genes (CD44, STAT3, CD47, ALDH1A1) in different molecular subtypes of MIBC. C and D, The expression levels of LASS2 in different molecular subtypes of breast cancer. *p < 0.05, determined by the Kruskal-Wallis test. **Fig. S3.** A, Left panel: Heatmap showing the correlation coefficient of each bladder cancer cell line for basal-like (BL) or non-basal-like (nBL) bladder cancer in GSE64572. Right panel: mRNA expression levels of CD44 in bladder cancer cell lines. B, qPCR analysis of CD44 expression in the indicated cell lines. C, Cisplatin IC_50_ vales of the indicated cell lines. D, Scatter plot showing the correlation between LASS2 expression levels and cisplatin IC_50_ values. r, Spearman correlation coefficient. E, The overexpression and knockdown efficiency of LASS2 was verified by western blot. F, Flow cytometry analysis of the percentage of CD44^+^ALDH1A1^+^ subpopulation in bladder cancer cell lines. G, Quantitative analysis of ceramides by LC–MS in chemoresistant and chemosensitive bladder cancer specimens. H, Pathway activity score of LASS2-high group versus LASS2-low group is presented though bubble color and size. The bubble color from green to red represents the pathway activity from inhibition to activation, and the bubble size is positively correlated with the FDR value. **Fig. S4.** A, Cell viability and cisplatin IC_50_ assay of cell lines from different cancer types. B, Table showing the cisplatin IC_50_ values in cell lines from different cancer types. C, Cell viability and IC_50_ assay of the 5637 cell line treated with different chemotherapy agents. **Fig. S5.** BCSC sorting and examination. A, The gating strategies of FACS sorting. B, Percentages of CD44^+^ALDH1A1^−^, CD44^−^ALDH1A1^+^, CD44^+^ALDH1A1^+^, and CD44^−^ALDH1A1^−^ subpopulations in BCSCs and BCNSCs. C, *In vitro* extreme limiting dilution assay of BCSCs and BCNSCs. D, qPCR analysis of CSC markers in BCSCs and BCNSCs. E-H, The indicated numbers (2×10^1^, 2×10^2^, 2×10^3^ and 2×10^4^) of BCSCs and BCNSCs were subcutaneously injected into nude mice. Tumor images (E), tumor incidence (F), and tumor weight (G, H) are shown. I, Quantitative analysis of ceramides by LC–MS in BCSCs and BCNSCs. J, Differentially expressed resistance-related genes in BCSCs were identified by an RT2 Profiler PCR Array (*p* < 0.05 and fold change > 5 genes were selected). Data are presented as the mean ± SD of at least three technical replicates. *p<0.05, **p<0.01, ***p<0.001, determined by Student’s t test. **Fig. S6.** A, Cisplatin sensitivity analysis of PDX tumors. The IC_50_ values of cisplatin for each tumor are presented in the plot. B, Percentage of CD44^+^ALDH1A1^+^ subpopulation in PDX tumors. C. Immunohistochemistry staining of CD44 in the indicated PDX tumors. **Fig. S7.** A, Measurement of mouse body weight during treatment. B, Hematological parameters of mice in different groups. C, Organ weight of mice in different groups. **Fig. S8.** Analysis of the CERS family genes. A, Heatmap showing the mRNA expression of CERS family genes in each cancer. B, qPCR analysis of CERS family genes in chemosensitive and chemoresistant bladder tumors. C, Pie plot showing the constitute of heterozygous/homozygous copy number variation (CNV) of CERS family genes in each cancer. D, Correlation analysis of CNV with mRNA. The mRNA expression data and CNV raw data were merged by TCGA barcode, followed by Spearman correlation analysis. Blue bubbles represent negative correlations, with red bubbles representing positive correlations. The bubble size is positively correlated with the FDR significance. The black outline border indicates FDR ≤ 0.05. **Fig. S9.** Original blot images.

**Additional file 3. **Materials and resources.

**Additional file 4. **PCR array.

**Additional file 5. **

**Additional file 6. **

**Additional file 7. **

**Additional file 8. **Pathway activity analysis.

## Data Availability

All data generated or analyzed during this study are available from the corresponding author on reasonable request.
