# Supplementary material for: LASS2 enhances chemosensitivity to cisplatin by inhibiting PP2A-mediated β-catenin dephosphorylation in a subset of stem-like bladder cancer cells
Source: BMC Med. 2024 Jan 9;22:19. doi: 10.1186/s12916-023-03243-5 (PMC10775422; doi:10.1186/s12916-023-03243-5)

## Supplementary Fig. S1

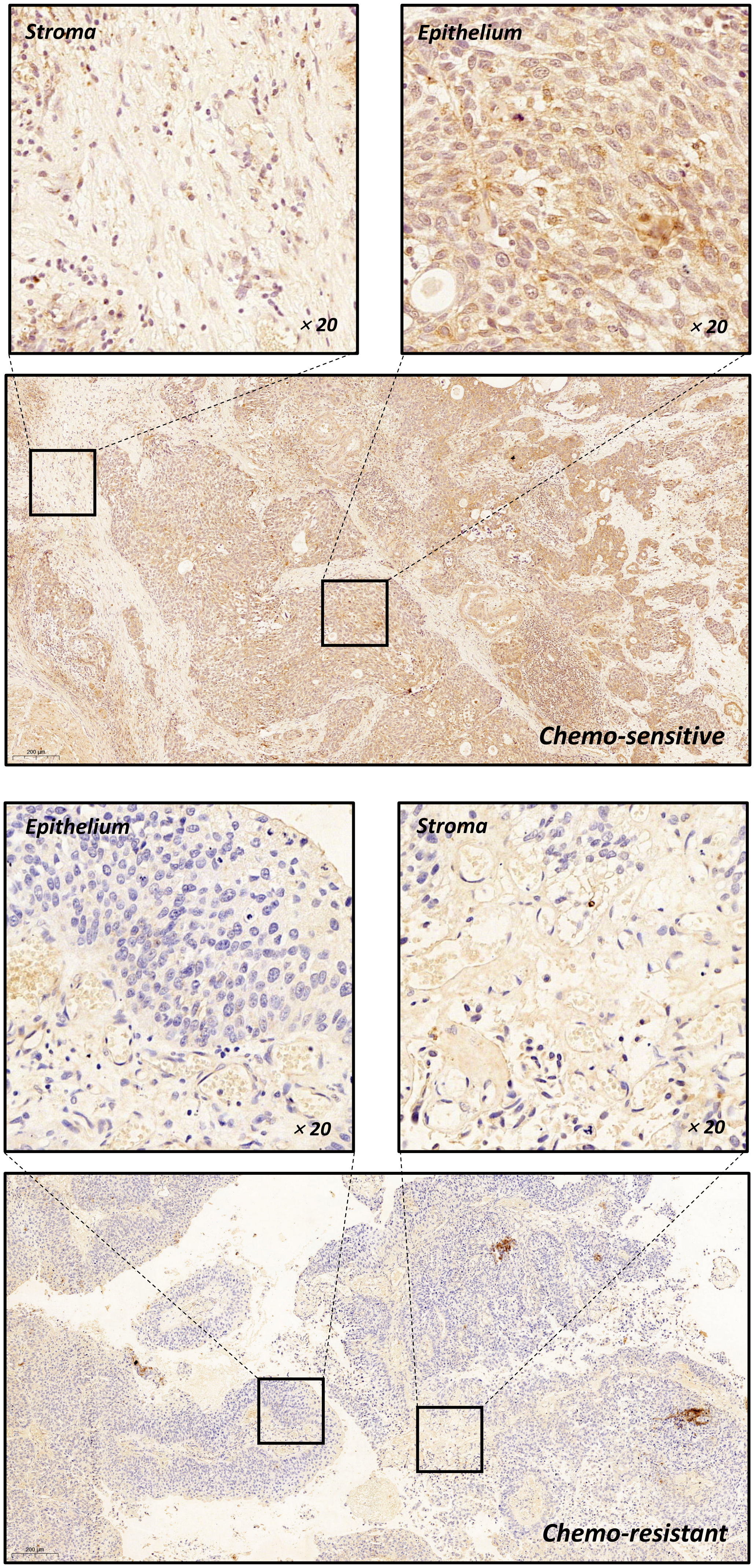

# Supplementary Fig. S2

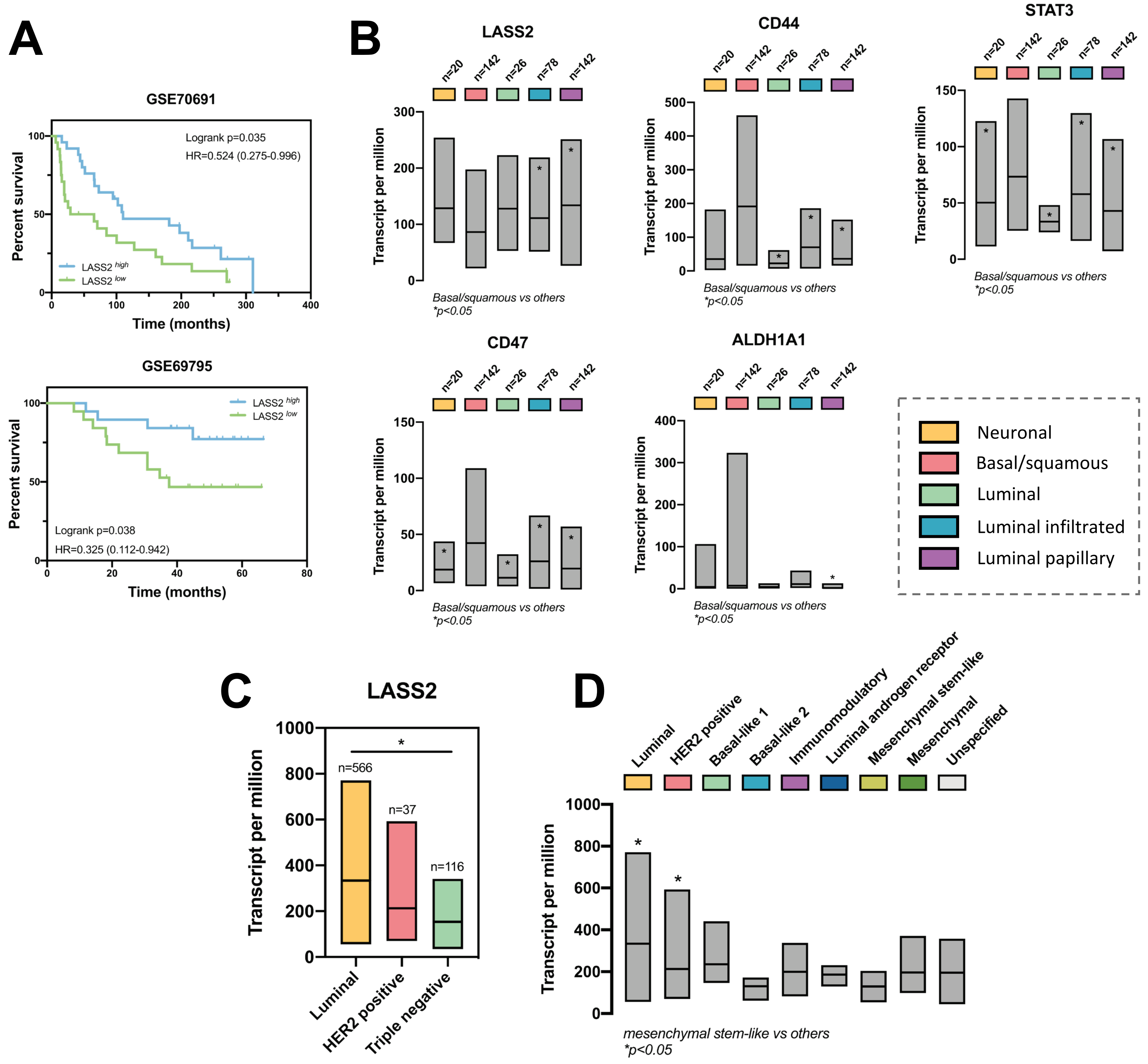

# Supplementary Fig. S3

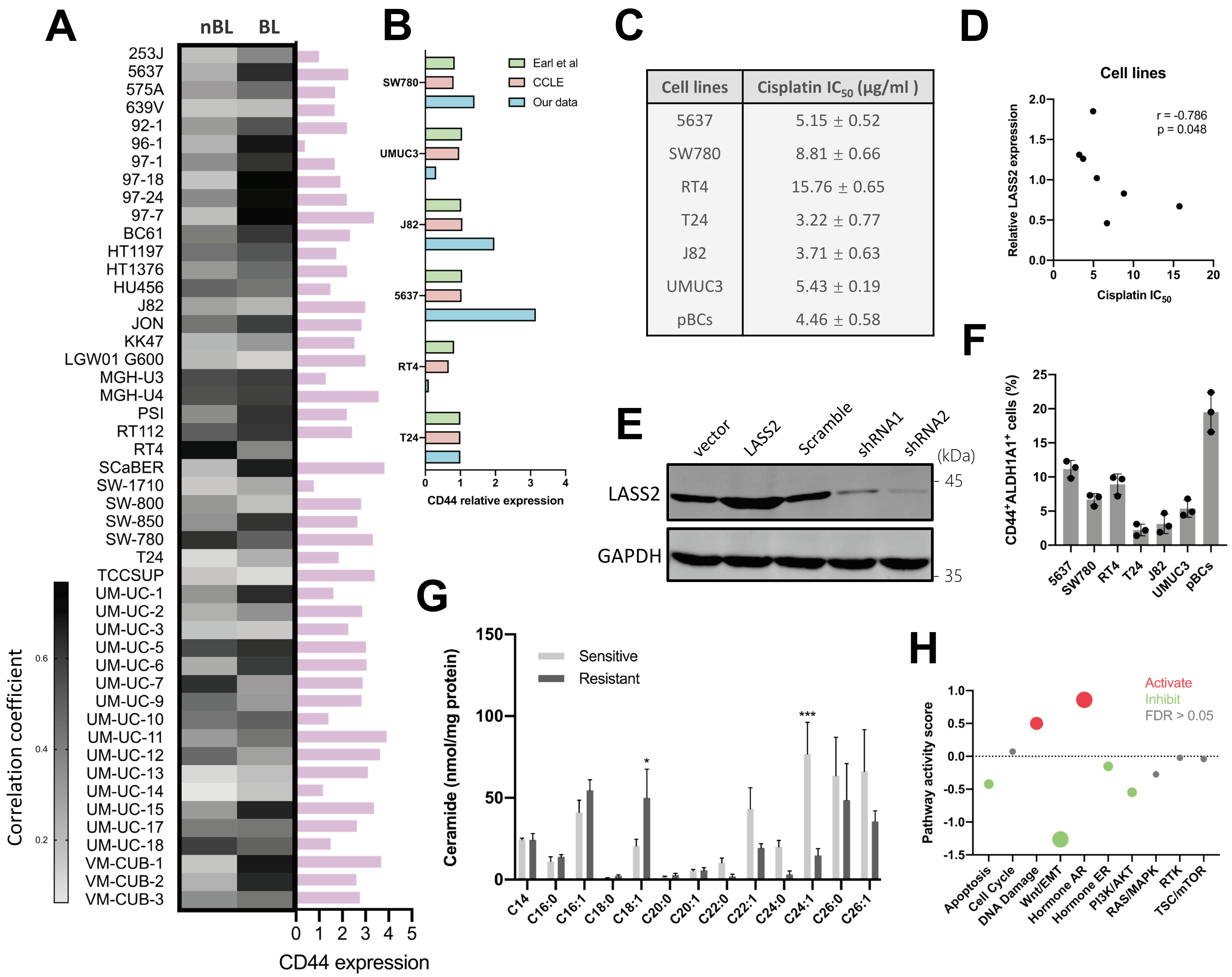

# Supplementary Fig. S4

A

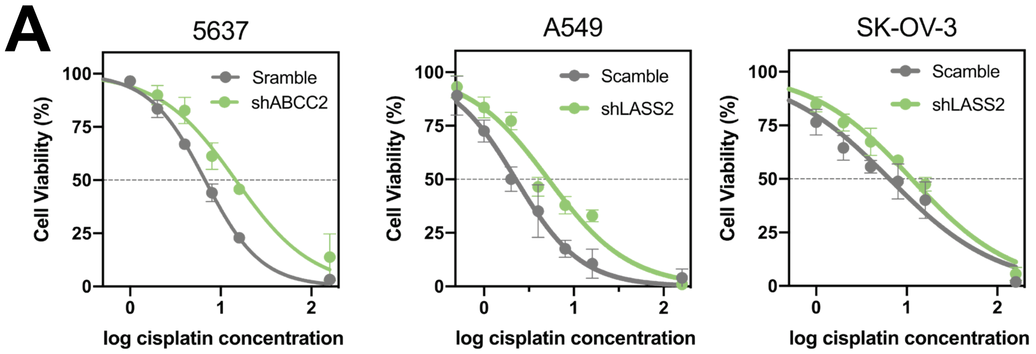


B

| Cell line | Cancer | Cisplatin IC$_{50}$ (μg/ml) | |
|---|---|---|---|
| | | Scramble | shLASS2 |
| 5637 | bladder cancer | 6.703 | 14.62 |
| A549 | lung cancer | 2.255 | 5.201 |
| Sk-OV-3 | ovarian cancer | 6.451 | 11.33 |

C

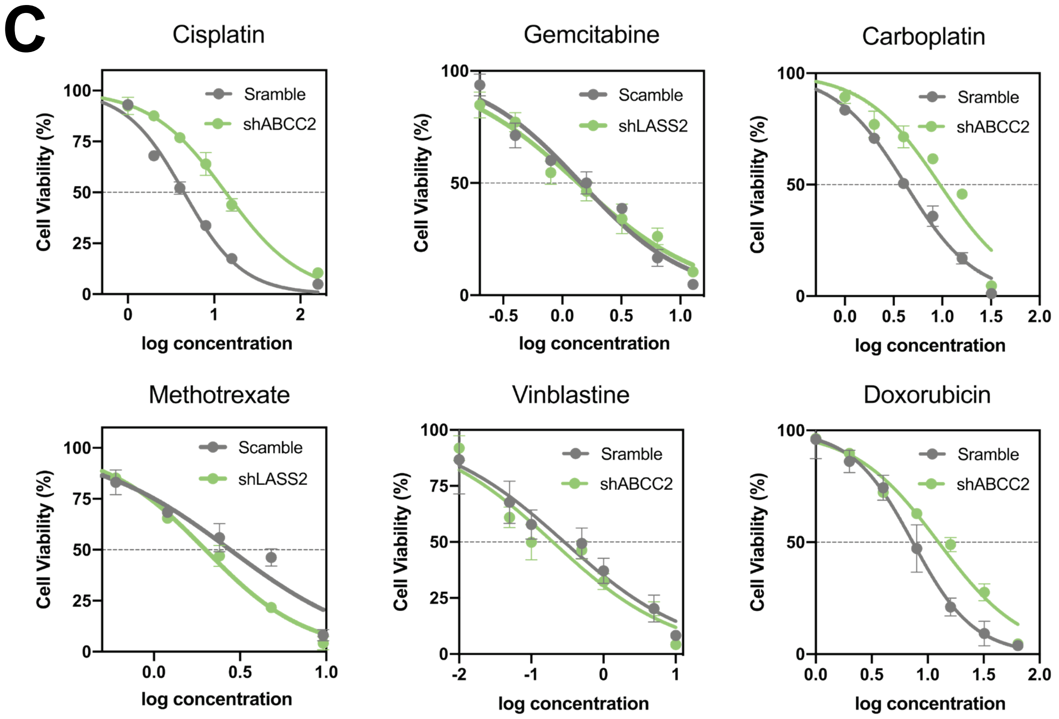

# Supplementary Fig. S5

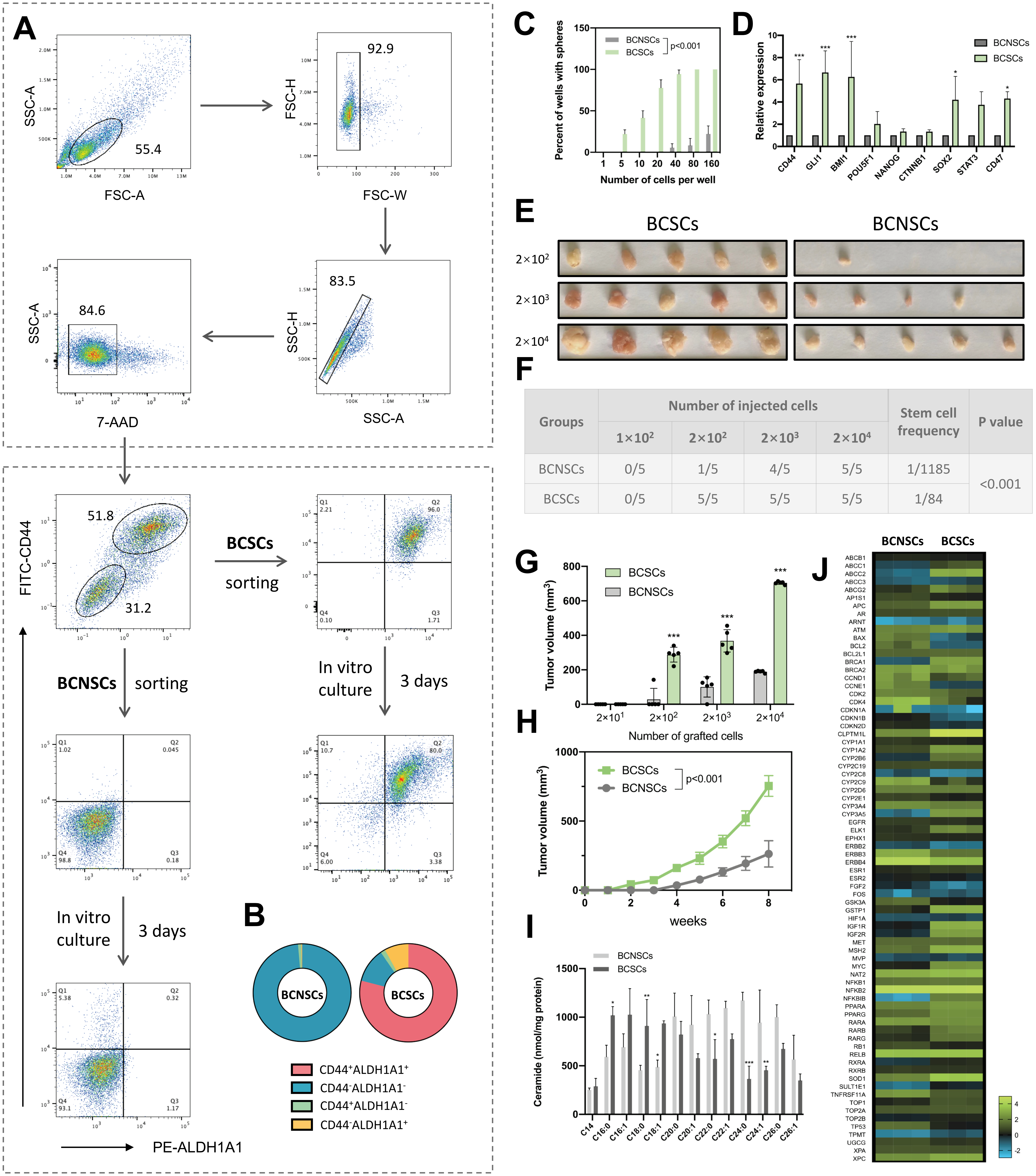


| Groups | Number of injected cells | | | | Stem cell frequency | P value |
|---|---|---|---|---|---|---|
| | $1\times10^2$ | $2\times10^2$ | $2\times10^3$ | $2\times10^4$ | | |
| BCNSCs | 0/5 | 1/5 | 4/5 | 5/5 | 1/1185 | <0.001 |
| BCSCs | 0/5 | 5/5 | 5/5 | 5/5 | 1/84 | |

## Supplementary Fig. S6

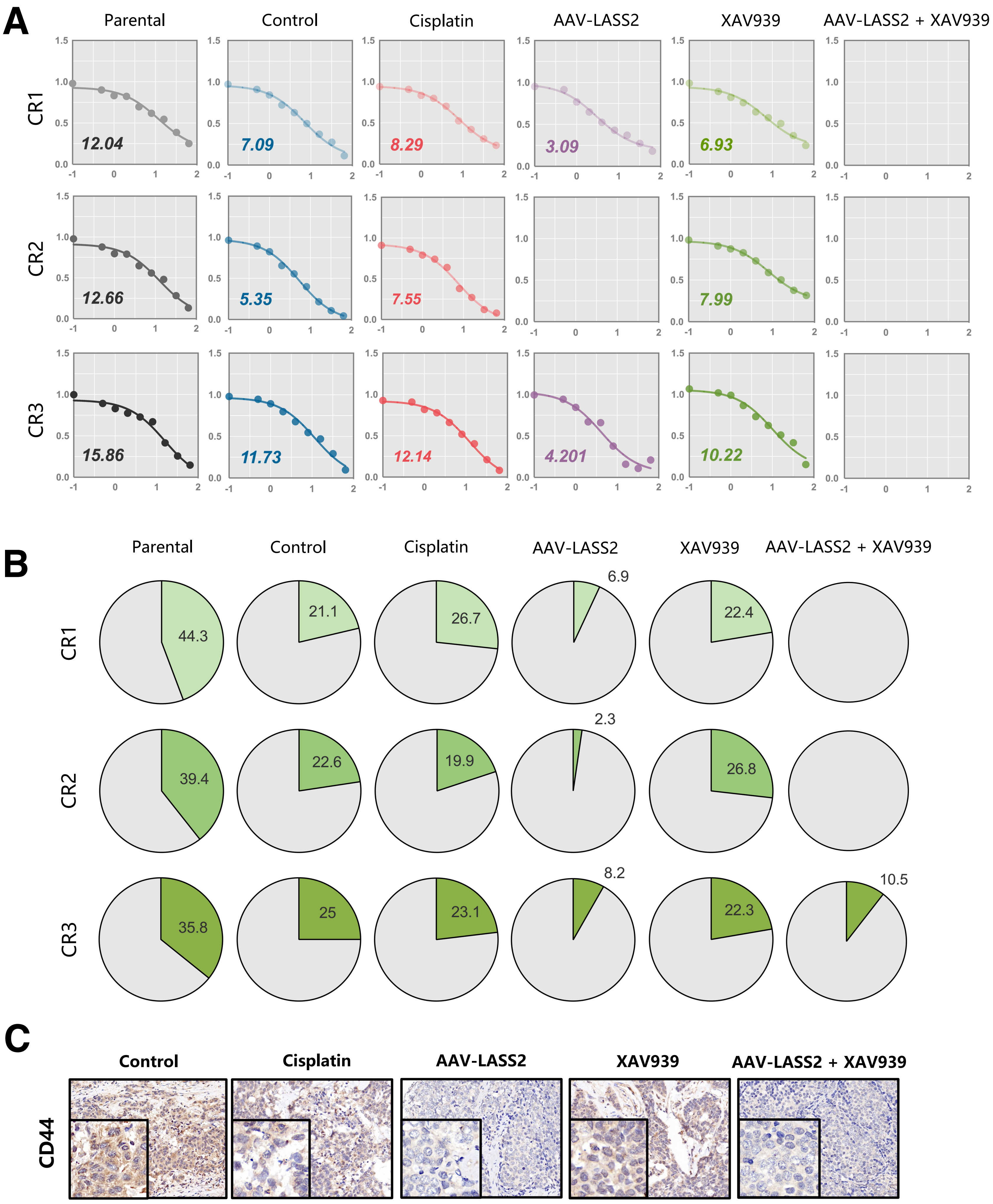

## Supplementary Fig. S7

A

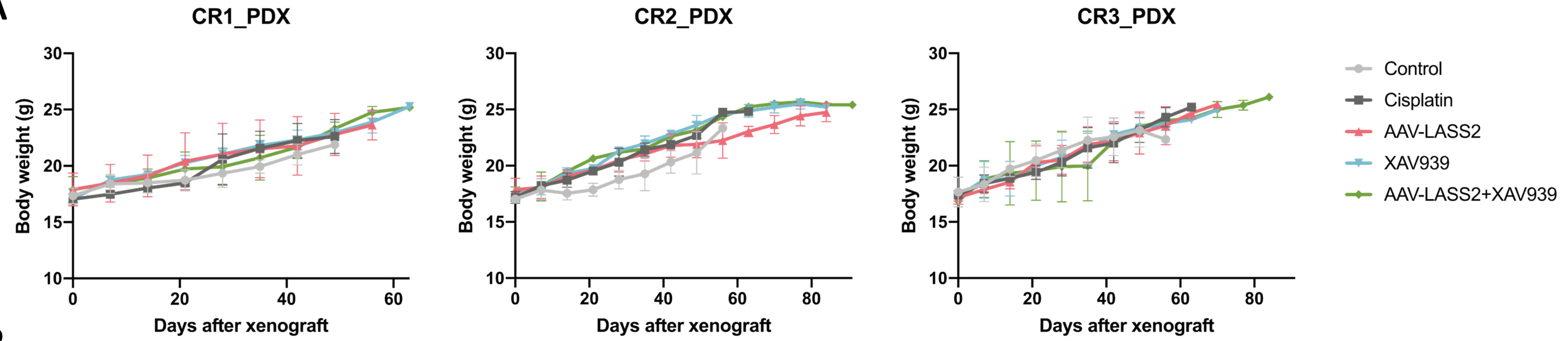


B

| Parameters | Normal range | Control | Cisplatin | Cisplatin AAV-LASS2 | Cisplatin XAV939 | Cisplatin AAV-LASS2 XAV939 |
|---|---|---|---|---|---|---|
| WBC (10^9/L) | 5.5-19.5 | 13.19 ± 3.07 | 4.00 ± 1.57 | 4.85 ± 1.54 | 5.56 ± 2.19 | 6.59 ± 4.07 |
| NEU (10^9/L) | 1.8-12.6 | 6.41 ± 2.72 | 3.94 ± 3.39 | 3.42 ± 3.69 | 5.39 ± 4.78 | 2.72 ± 2.59 |
| LYM (10^9/L) | 0.8-7.9 | 3.55 ± 1.81 | 0.39 ± 0.21 | 0.79 ± 0.43 | 0.99 ± 0.96 | 1.44 ± 1.50 |
| MON (10^9/L) | 0-1.8 | 0.62 ± 0.85 | 0.23 ± 0.24 | 0.69 ± 0.65 | 0.67 ± 0.52 | 0.43 ± 0.45 |
| RBC (10^12/L) | 5.1-11.2 | 8.85 ± 1.16 | 5.70 ± 1.47 | 5.99 ± 1.59 | 6.91 ± 1.54 | 5.50 ± 1.79 |
| HCT (%) | 26-51 | 34.94 ± 8.58 | 29.15 ± 4.64 | 29.56 ±4.97 | 31.85 ± 11.70 | 30.00 ± 10.74 |
| MCV (fL) | 35-54 | 36.61 ± 10.56 | 40.64 ± 6.21 | 42.93 ± 7.28 | 40.54 ± 5.62 | 38.01 ± 3.00 |
| HGB (g/L) | 85-162 | 143.63 ± 21.07 | 97.38 ± 29.70 | 99.13 ± 20.60 | 114.13 ± 22.60 | 94.86 ± 30.33 |
| PLT (10^9/L) | 100-518 | 296.63 ± 155.33 | 92.00 ± 17.88 | 140.25 ± 89.523 | 112.50 ± 102.01 | 93.57 ± 43.94 |
| PCT (%) | 0.09-0.7 | 0.37 ± 0.17 | 0.18 ± 0.08 | 0.32 ± 0.26 | 0.31 ± 0.19 | 0.26 ± 0.18 |
| MPV (fL) | 8.2-16.3 | 12.64 ± 3.35 | 8.21 ± 1.97 | 8.13 ± 2.50 | 7.86 ± 3.43 | 9.34 ± 2.03 |

C

| Organs | Control | Cisplatin | Cisplatin AAV-LASS2 | Cisplatin XAV939 | Cisplatin AAV-LASS2 XAV939 |
|---|---|---|---|---|---|
| Liver | 2.05 ± 0.38 | 1.56 ± 0.59 | 1.44 ± 0.07 | 1.51 ± 0.10 | 1.39 ± 0.19 |
| Lung | 0.65 ± 0.23 | 0.70 ± 0.22 | 0.50 ± 0.13 | 0.55 ± 0.20 | 0.61 ± 0.38 |
| Kidney | 0.40 ± 0.18 | 0.32 ± 0.18 | 0.38 ± 0.22 | 0.34 ± 0.06 | 0.31 ± 0.09 |
| Heart | 0.23 ± 0.08 | 0.24 ± 0.06 | 0.21 ± 0.04 | 0.31 ± 0.34 | 0.23 ± 0.11 |
| Spleen | 0.07 ± 0.01 | 0.06 ± 0.02 | 0.06 ± 0.01 | 0.17 ± 0.32 | 0.07 ± 0.02 |
| Brain | 0.41 ± 0.06 | 0.40 ± 0.06 | 0.41 ± 0.08 | 0.41 ± 0.04 | 0.38 ± 0.07 |
| Testis | 0.19 ± 0.03 | 0.19 ± 0.04 | 0.19 ± 0.03 | 0.29 ± 0.26 | 0.30 ± 0.32 |

## Supplementary Fig. S8

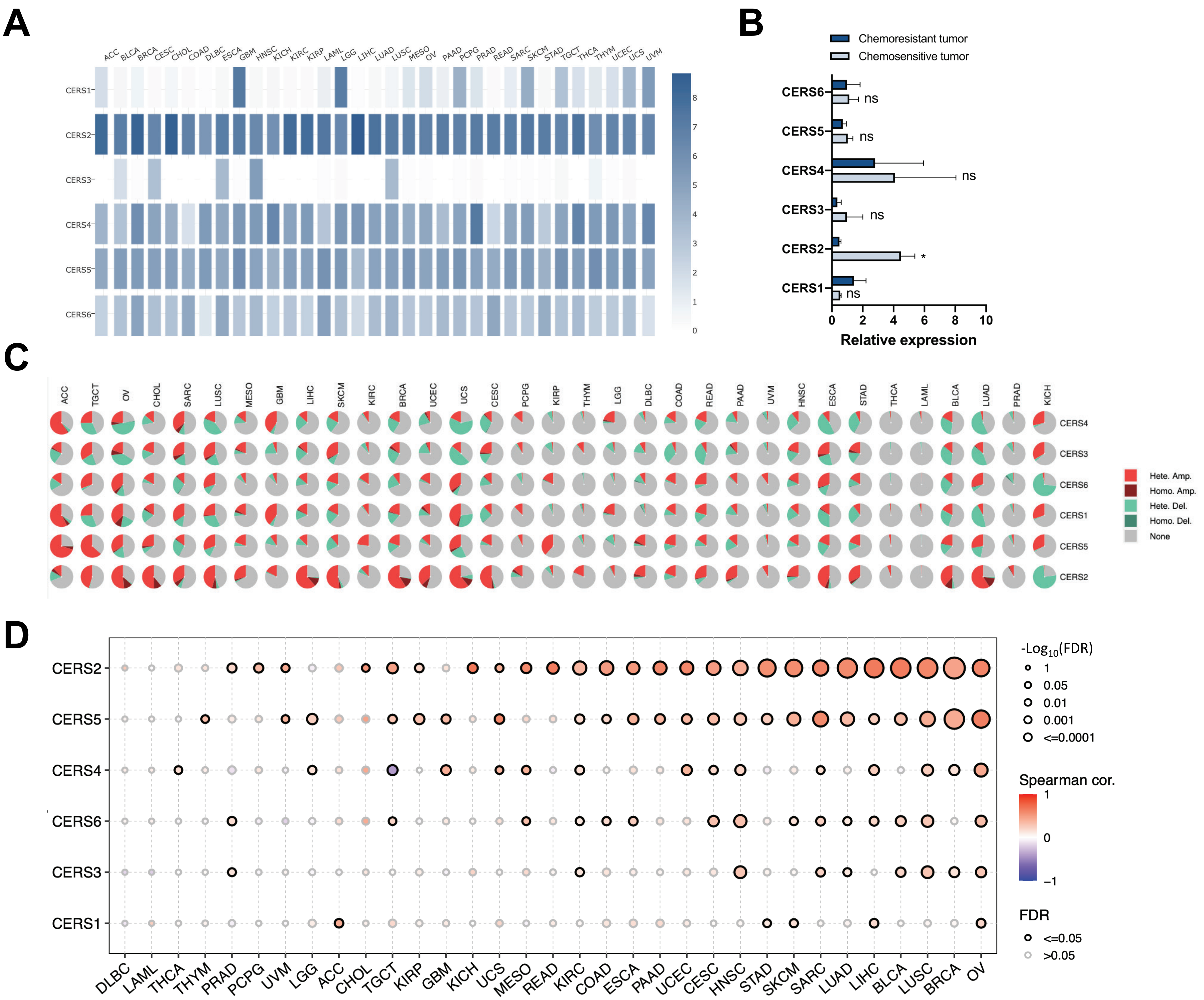

# Supplementary Fig. S9

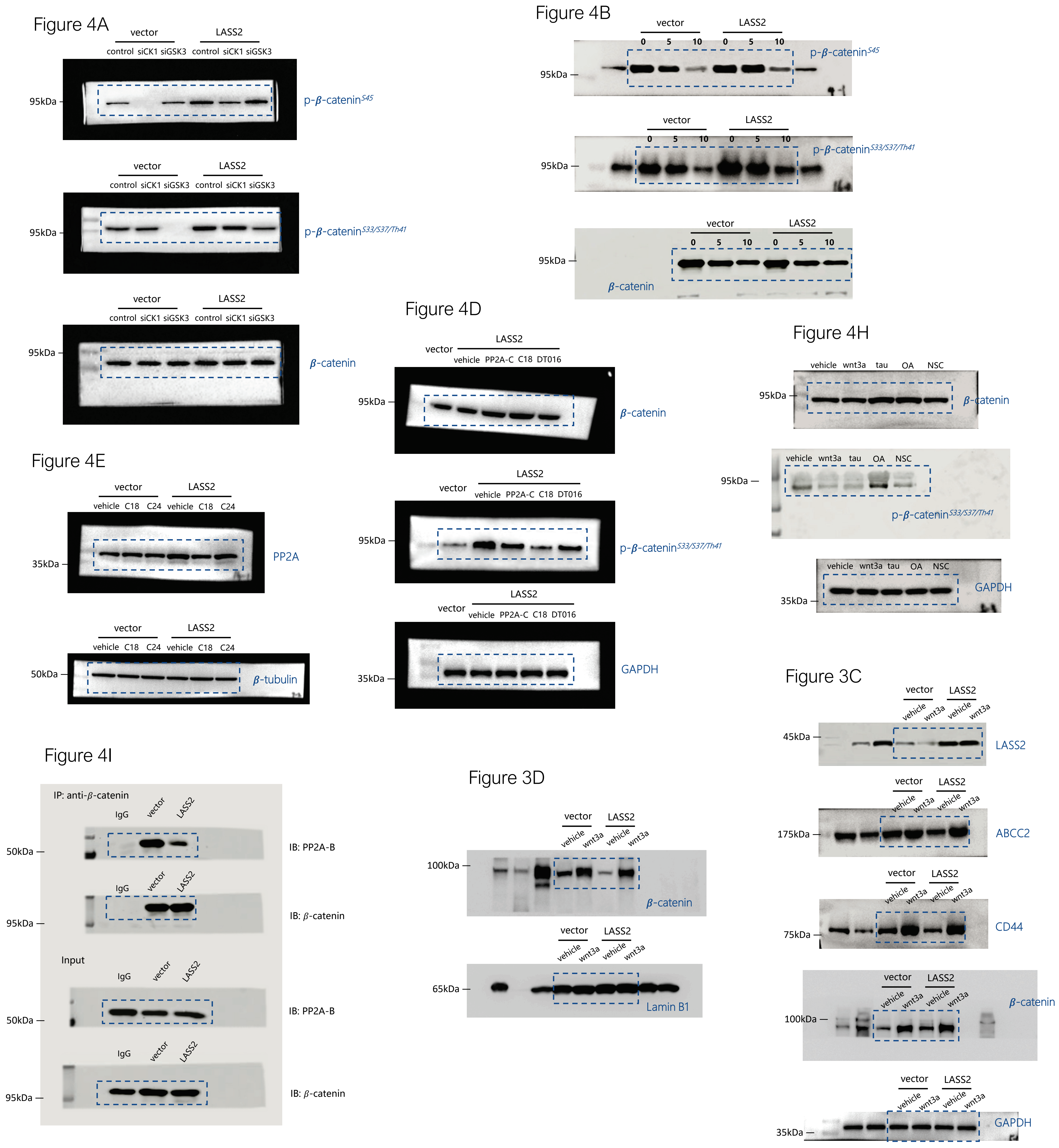

Supplement: Supplementary file 2 — Additional file 2: Fig. S1. High-resolution images of immunohistochemical staining. Fig. S2. A, Kaplan–Meier curve and Log-rank test of overall survival for patients received cisplatin-based chemotherapy in GSE70691 and GSE69795 datasets. B, The expression levels of LASS2 and stemness-related genes (CD44, STAT3, CD47, ALDH1A1) in different molecular subtypes of MIBC. C and D, The expression levels of LASS2 in different molecular subtypes of breast cancer. *p < 0.05, determined by the Kruskal-Wallis test. Fig. S3. A, Left panel: Heatmap showing the correlation coefficient of each bladder cancer cell line for basal-like (BL) or non-basal-like (nBL) bladder cancer in GSE64572. Right panel: mRNA expression levels of CD44 in bladder cancer cell lines. B, qPCR analysis of CD44 expression in the indicated cell lines. C, Cisplatin IC50 vales of the indicated cell lines. D, Scatter plot showing the correlation between LASS2 expression levels and cisplatin IC50 values. r, Spearman correlation coefficient. E, The overexpression and knockdown efficiency of LASS2 was verified by western blot. F, Flow cytometry analysis of the percentage of CD44+ALDH1A1+ subpopulation in bladder cancer cell lines. G, Quantitative analysis of ceramides by LC–MS in chemoresistant and chemosensitive bladder cancer specimens. H, Pathway activity score of LASS2-high group versus LASS2-low group is presented though bubble color and size. The bubble color from green to red represents the pathway activity from inhibition to activation, and the bubble size is positively correlated with the FDR value. Fig. S4. A, Cell viability and cisplatin IC50 assay of cell lines from different cancer types. B, Table showing the cisplatin IC50 values in cell lines from different cancer types. C, Cell viability and IC50 assay of the 5637 cell line treated with different chemotherapy agents. Fig. S5. BCSC sorting and examination. A, The gating strategies of FACS sorting. B, Percentages of CD44+ALDH1A1−, CD44−ALDH1A1+, CD4 [file 12916_2023_3243_MOESM2_ESM.pdf]
